# Endoplasmic reticulum patterns insect cuticle nanostructure

**DOI:** 10.1083/jcb.202503127

**Published:** 2025-12-29

**Authors:** Sachi Inagaki, Housei Wada, Takeshi Itabashi, Yuki Itakura, Reiko Nakagawa, Lin Chen, Kazuyoshi Murata, Atsuko H. Iwane, Shigeo Hayashi

**Affiliations:** 1 Laboratory for Morphogenetic Signaling, RIKEN Center for Biosystems Dynamics Research, Kobe, Japan; 2 Laboratory for Cell Field Structure, RIKEN Center for Biosystems Dynamics Research, Kobe, Japan; 3 Department of Molecular and Cellular Physiology, Graduate School of Medicine, Yamaguchi University, Ube, Japan; 4 Laboratory for Cell-Free Protein Synthesis, RIKEN Center for Biosystems Dynamics Research; 5 Exploratory Research Center on Life and Living Systems (ExCELLS) and National Institute for Physiological Sciences (NIPS), National Institutes of Natural Sciences, Okazaki, Japan; 6 Department of Physiological Sciences, School of Life Science, The Graduate University for Advanced Studies (SOKENDAI), Okazaki, Japan; 7 Division of Advanced Genome Editing Therapy, Research Institute for Cell Design Medical Science, Yamaguchi University, Ube, Japan; 8 Department of Biology, Kobe University Graduate School of Science, Kobe, Japan

## Abstract

Insect cuticles with nano-level structures exhibit functional surface properties such as the photonic nanocrystal of the butterfly wing scale with structural color and the corneal nipple arrays of superhydrophobic compound eye lens. Despite the enormous influence the cuticle has had on biomimetic industrial applications, cellular mechanisms of cuticular nanopatterning remain poorly understood. *Drosophila gore-tex/Osiris23 (gox)* controls the formation of nanopores, with a molecular filtering function, on the olfactory organs. Here we used 3D electron microscopy imaging of entire hair structures to show that nanopore is formed through a novel process of bidirectional interaction of the ER and the plasma membrane trafficking. ER-resident protein Gox stimulates ER-phagy through regulation of Ref(2)P, the fly counterpart of the autophagy protein p62/SQSTM1, and initiates endocytosis. Dynamin on the plasma membrane completes endocytosis and sustains ER-phagy. The repurposing of ER-phagy for plasma membrane remodeling and the fabrication of nanoscale ECM structures sheds light on the nanopatterning mechanism of insect cuticles and their genetic control.

## Teaser

An extensive ER network develops in the olfactory hair cells of *Drosophila*. Inagaki et al. report that the transmembrane protein Gore-tex controls a novel ER to the plasma membrane trafficking pathway essential for the cuticular nanopore formation.

## Introduction

Nanoscale modifications of the insect cuticle are the basis for various surface properties, such as efficient light transmittance and molecular filtering by sensory organs, and the selective light reflection that underlies intra- and inter-specific communication (Blagodatski et al., 2015; Kryuchkov et al., 2020; Thayer and Patel, 2023; Steinbrecht, 1997). These key structural innovations have enabled ancestral insects to explore and thrive in a wide range of terrestrial environments. The insect cuticle is a multilayered structure of great variety in thickness and chemical composition (Wigglessworth, 1948; Chapman, 2012). While the biochemical basis for the unique physical properties of cuticles has been investigated, how cuticular nanostructures are patterned at the cellular and genetic levels is poorly known. Ghiradella (1989) described how a crystallin-like lattice of ER prefigures the patterning of the porous cuticle of the butterfly wing scale with structural color (Ghiradella, 1989). However, the role of intracellular organelles in cuticular ECM patterning has not been addressed so far.

**Figure 1.**
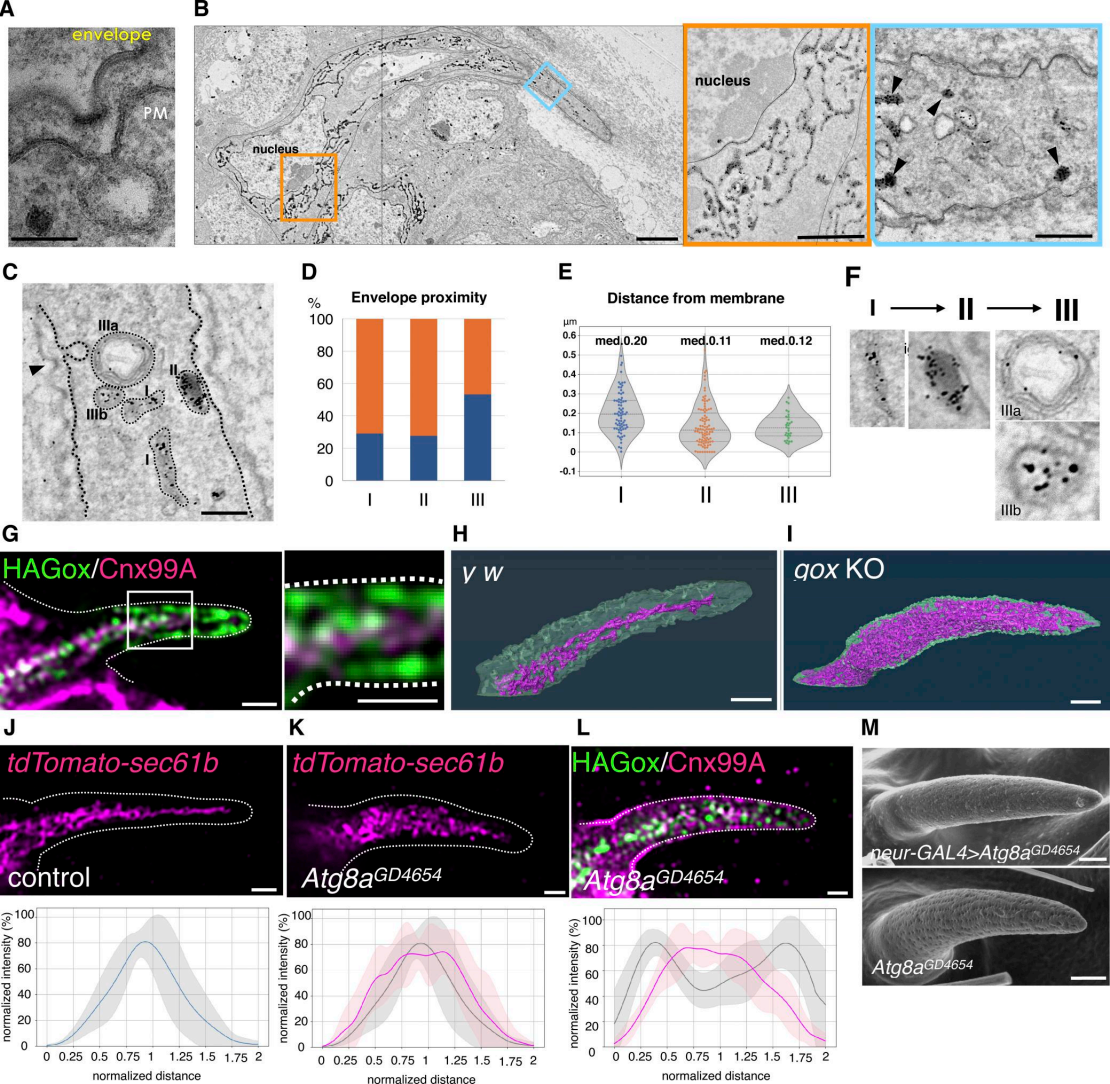
**ER-localized Gox/Osi23 controls ER-phagy. (A–C)** TEM of the olf hair cell at 44 h APF. **(A)** An enlarged view of the curved cuticular envelop and the underlying plasma membrane. The lowest point of the curved envelope is often associated with PMI. **(B)** Localization of APEX2-Gox protein in tubular ER. Left: low-magnification view (composite of two images). Right: enlarged view of the perinuclear (orange) and the shaft region (blue). Dotted lines outline plasma membrane of the olf hair cell. **(C)** Three types of Gox-containing organelles. I, ER. II, electron-dense vesicle (arrowhead in B). IIIa, IIIb, electron-lucent structures. IIIa is a multi-membrane structure. **(D)** Proximity of Gox+ structures to the high **(orange) **or low** (blue)** point of the envelope. **(E)** Distance from the membrane of the three types of Gox vesicles. Median distance is shown above each plot. **(F)** Presumed order of Gox-vesicle conversion. **(G)** Localization of Gox and ER marker Cnx. **(H and I)** Distribution of ER (magenta) in control (y w) and gox mutant. Segmented view of FIB-SEM stacks. **(J and K)** ER distribution in control and ATG8a RNAi. **(L)** Reduction of subcortical Gox localization in ATG8a RNAi. Graphs below show averaged line scan intensities of ER or Gox along the cross section of each hair (control: gray; mutant: magenta, 5–7 hairs for each genotype). **(M)** Adult olf bristle of neur > ATG8a RNAi lacking nanopores (top) and control (UAS-ATG8a RNAi only, bottom). For panel E, a total of 196 Gox-positive vesicles (type I = 72, type II = 94, and type III = 30) were classified by morphology and distance from the plasma membrane. The high- and low-PM curvature points were manually identified. No formal statistical test was applied because the data represent categorical frequency counts. For panels J–L, line intensity profiles of ER and HA–Gox signals were obtained from 5–7 individual hairs per genotype, normalized, and averaged using custom Python scripts. No formal statistical test was applied; error shading represents mean ± SD. Bar: 100 nm (A), 2 µm (B), 500 nm (enlarged in B), 200 nm (C), and 1 µm (G–M).

The *Osiris (Osi)* gene family includes multiple candidate regulators of such nanoscale patterning. Molecular phylogeny has shown that these genes were acquired early in insect evolution, rapidly increasing in number and becoming durably conserved thereafter (Dorer et al., 2003; Shah et al., 2012). In *Drosophila*, many of the 25 *Osi* genes are expressed in a variety of cuticle-secreting cells. Specific subsets of *Osi* genes are required for the cuticular patterning of the corneal nipple of the compound eye *(Osi9* and *Osi21)*, the tip pore of the taste hair *(Osi11)*, and nanopores of olfactory (olf) hairs (*gore-tex/Osi23* (*gox*) (Ando et al., 2019; Sun et al., 2024)). Nanopores are 30–50-nm pores on the surface cuticles of sensilla basiconica and sensilla trichordia, which function in the selective transport of airborne odorant molecules to olf neurons while excluding larger particles (Steinbrecht, 1997; Shanbhag et al., 1999). Nanopore development starts at the earliest stage of cuticle development as the wavy curving of the envelope layer, which tracks the curved and invaginated patterns of the plasma membrane in olf hair cells (Ando et al., 2019). *gox* is required for maintaining the curvature of the plasma membrane, which underlies nanopore formation and proper olf response (Ando et al., 2019). Notably, ∼70% of the membranes showing endocytic structures are proximal to a downward curved envelope (Fig. 1 A), leading to the hypothesis that the plasma membrane serves as a template for curved envelope formation (Ando et al., 2019). Gox is known as a transmembrane protein localized to intramembrane compartments, but its function in the plasma membrane and cuticular nanostructures is not well understood.

We now show the molecular pathway by which Gox exerts its patterning functions. Gox is localized to the ER and triggers ER-phagy by recruitment of the autophagy mediator Ref(2)P (Nagai et al., 2021), a homolog of mammalian p62/SQSTM1 (Chino and Mizushima, 2020). The processed ER-phagy product gains access to the plasma membrane and promotes plasma membrane invagination (PMI). Invaginated plasma membranes are cleared through the excision by dynamin, which also stimulates ER-phagy. Those interactions form positive feedback of sustained ER-phagy and plasma membrane remodeling. Thus, in insects, the Osi-family protein Gox has accepted the conserved ER-phagy pathway for the plasma membrane remodeling underpinning the nanoscale patterning of apical ECM.

## Results

### Gox promotes ER-phagy during nanopore formation

Gox has been localized to intracellular vesicles that overlap endosomal markers (Ando et al., 2019). To determine the precise identity of the Gox-carrying organelle, we knocked the APEX2 tag (Martell et al., 2017) into the N terminus of the genomic *gox* gene. The tag, which did not affect Gox activity, allowed the labeling of the protein in transmission electron microscopy (TEM). APEX2 signal appeared in the luminal area of tubular ER structures (type I) surrounding the nucleus and in the distal shaft region of the olf cells (sensilla basiconica of the 3rd antennal segment, An3, Fig. 1, B and C). Additional labels were found in electron-dense vesicles (type II), multi-membrane structures (type IIIa), and vesicles with opaque staining (type IIIb, Fig. 1 C). Type II vesicle had the highest level of Gox and was located most proximal to the plasma membrane (Fig. 1, C and E). Type IIIa and type IIIb structures were sometimes located adjacent to invaginated membranes and curved envelopes (Fig. 1, C and D). A type I–type II –type III transition was deduced from the assumption of a linear relationship (Fig. 1, E and F). Consistently, fluorescently labeled HA–Gox overlapped partially with the tubular ER network in the central region of the olf cell and was densely localized underneath the plasma membrane, likely in type II vesicles (Fig. 1 G). This localization pattern indicates Gox is transported from ER into membrane-proximal structures.

**Figure S1.**
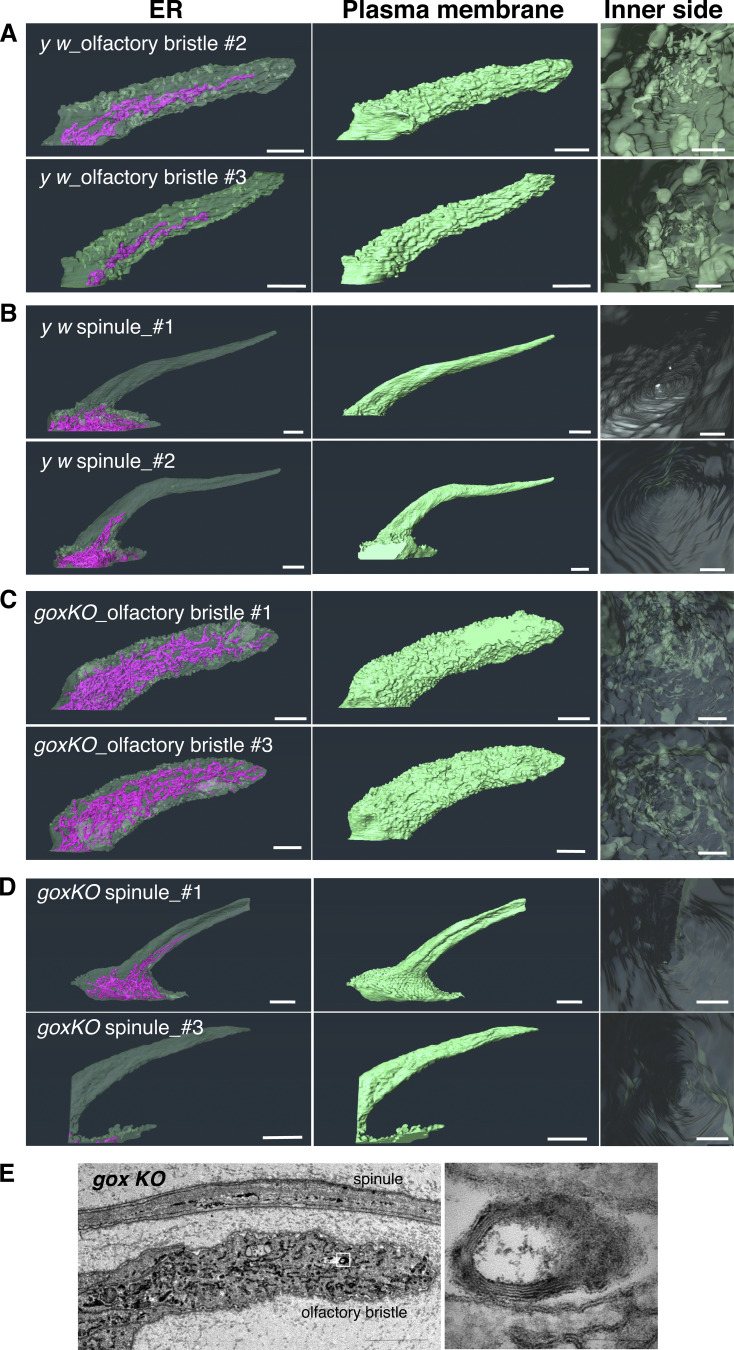
**FIB-SEM reconstructions of ER and plasma membrane in multiple hair cells of olf and spinule. **Sampled from the An3 at 42 h APF. An enlarged view of the internal side of the plasma membrane is shown on the right.** (A)** Control olf. **(B)** Control spinule. **(C)***gox*^*1*^ olf. **(D)***gox*^*1*^spinule. **(E)** TEM view of the *gox* mutant olf. ER whorl structure is enlarged. Bar: 1 µm. **(A–D)** 200 nm (A–D PM inner side, approx.), 2 µm (E left), and 100 nm (E enlarged).

In the olf hair cell, the ER formed an interconnected network extending along the cell’s major axis (Fig. 1 H and Fig. S1 A). ER network formation was poor in the non-olf hair (spinule, Fig. S1 B) In *gox* mutants, ER overexpanded to fill the entire cytoplasm of the olf hair cell (Fig. 1 I and Fig. S1 C), but not the spinule (Fig. S1 D). TEM of *gox* mutant olf hair cells revealed vacuolar structures with multilayered membranes, similar to the ER whorls observed in ER-stressed cells (Feldman et al., 1981; Bernales et al., 2006) (Fig. S1 E).

**Figure S2.**
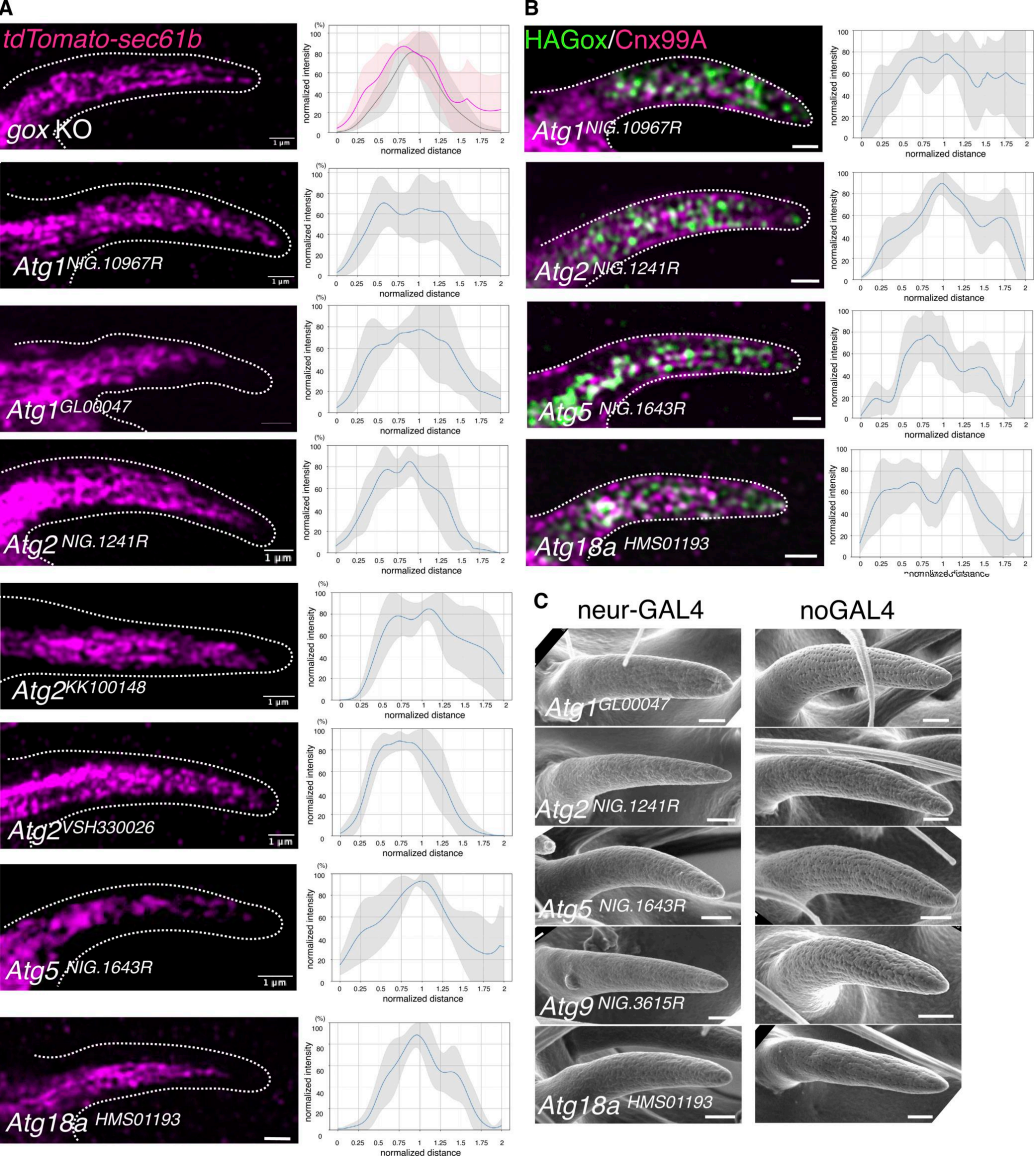
**Knockdown of the ATG genes causes ER overexpansion, Gox mislocalization, and nanopore loss. (A)** ER distribution in *gox* mutants, and ATG1, Atg2, Atg5 and ATG18a RNAi. Graphs in the right show averaged line scan intensities of ER or Gox along the cross section of each hair (5–7 hairs for each genotype). Line intensity profiles were obtained using the *PlotProfile* function in *Fiji* and averaged after normalization with custom Python scripts. In the *gox* KO panel (top), *gox* mutant scan is shown in magenta, and control scan in gray. For other graphs, only mutant scan data are shown. **(B)** HA–Gox distribution in ATG1, ATG2, ATG5, and ATG18a RNAi showing the reduction of subcortical Gox. **(C)** olf hairs of ATG RNAi experiments. All showed reduced nanopores compared with the control. Bar: 1 µm.

The bulk amount of ER is negatively regulated by ER-phagy under control of the autophagy pathway (Chino and Mizushima, 2020; Rudinskiy and Molinari, 2023). Knockdown of the core autophagy genes (ATG1, ATG2, ATG5, ATG8a, and ATG18a) caused expansion of ER and concomitant reduction of Gox from the membrane-proximal region (Fig. 1, J–L; and Fig. S2, A and B). Mutant flies showed reduced nanopore formation (Fig. 1 M and Fig. S2 C), suggesting that ER-phagy driven by Gox is essential to nanopore formation.

### PMI is essential for the nanopore formation

**Figure 2.**
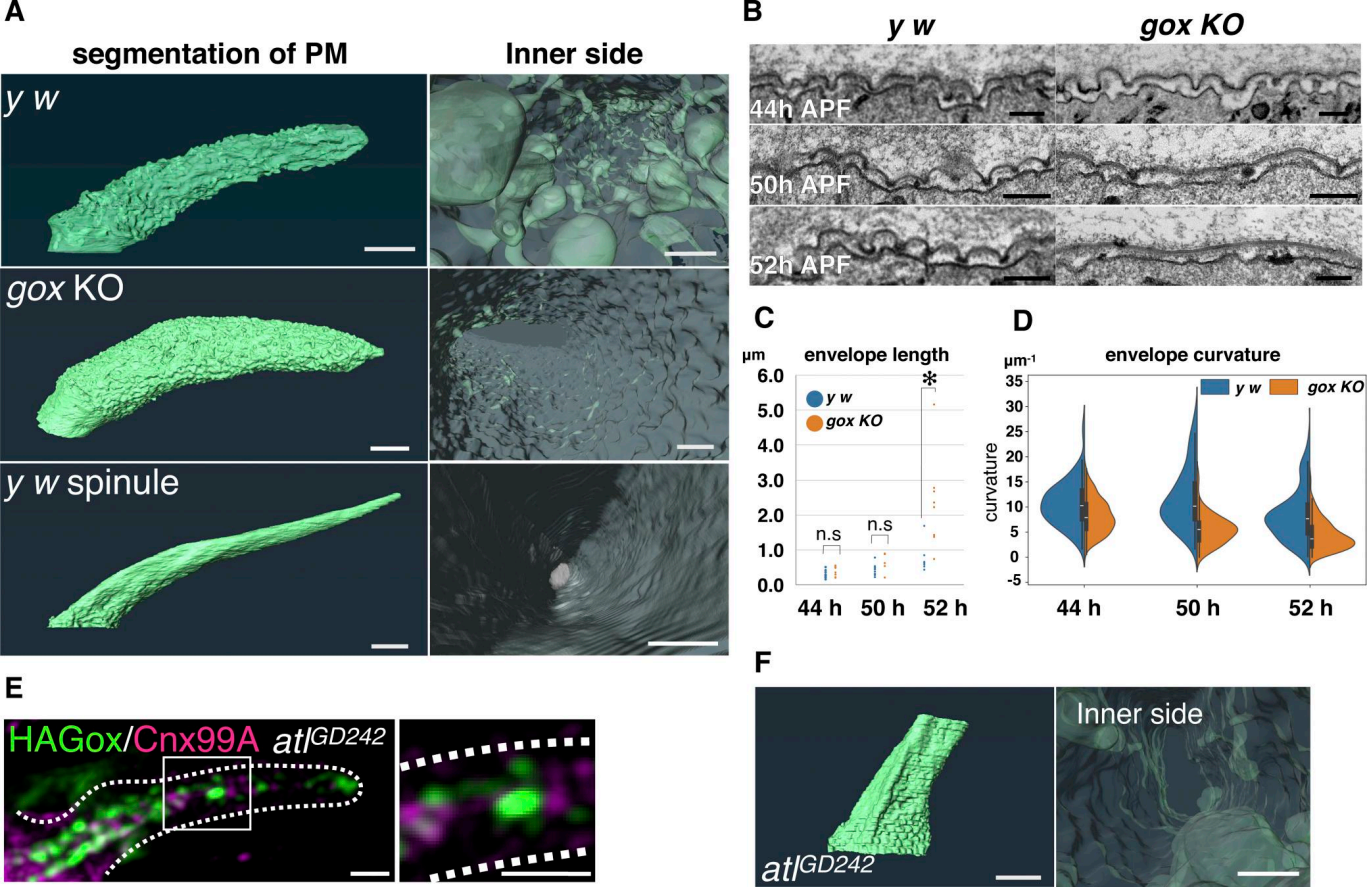
**Plasma membrane landscape of the olf hair cell. (A)** Plasma membrane structures of the olf and spinule obtained by FIB-SEM. Outer view (left) and inner view (right). **(B)** Longitudinal TEM views of the plasma membrane and envelope at 44, 50, and 52 h APF. **(C)** Envelope lengths were measured between adjacent low points in TEM images. Data points represent individual envelope fragments from *n* = 1–3 biological replicates per time point. A total of 95–183 fragments (5–9 TEM sections per condition) were analyzed. Data are shown as mean ± SD, and statistical significance between y w and gox KO was determined by the Mann–Whitney U test (two-sided). *: P < 0.05. n.s., not significant. **(D)** Quantification of envelope curvature (µm-1). Median point curvature per fragment was plotted in violin plots (*n* = 1–3 biological replicates). Statistical comparison used the Mann–Whitney U test (two-sided). **(E)** ER and Gox distributions in atl RNAi. **(F)** FIB-SEM plasma membrane views of *atl* RNAi hair cell #1. Bar: 1 μm (A, E, and F), 200 nm (B and E enlarged), and 200 nm (A and F inner side, approx.).

To study the mechanics of plasma membrane patterning by Gox, we performed a 3D reconstruction of the plasma membrane using focused ion beam-scanning electron microscopy (FIB-SEM [Heymann et al., 2006]). Olf hair cells showed a highly convoluted plasma membrane (plasma membrane convolution, PMC) on observation from the outside, whereas the inside view showed numerous plasma membrane invaginations (PMI, 74 per hair, *N* = 3, Fig. 2 A, control y *w*, and Fig. S1 A and Video 1). In *gox* mutants, the size of PMI was greatly reduced, while PMC was retained (Fig. 2 A, middle row, and Fig. S1 C). In contrast, spinules showed smooth membrane surfaces both outside and inside (Fig. 2 A, bottom row, and Fig. S1, B and D). These findings suggest that *gox* is involved in PMI formation. Time course analysis showed the envelope in *gox* mutants is curved at 44 h APF, gradually becoming flattened by 52 h APF (Fig. 2, B–D).

**Video 1.** **3D view of the plasma membrane of the olf hair cell #2 reconstructed from a segmented FIB-SEM stack.** A 3D stack of SEM images was acquired with a Helios G4 UC (Thermo Fisher Scientific), and image segmentation and 3D reconstruction were performed with Amira (Thermo Fisher Scientific). The movie starts from the external view, showing a highly convoluted plasma membrane. It proceeds to the internal view, travelling from the base to the tip, showing numerous invaginated plasma membranes, highlighted in lighter color.

**Figure S3.**
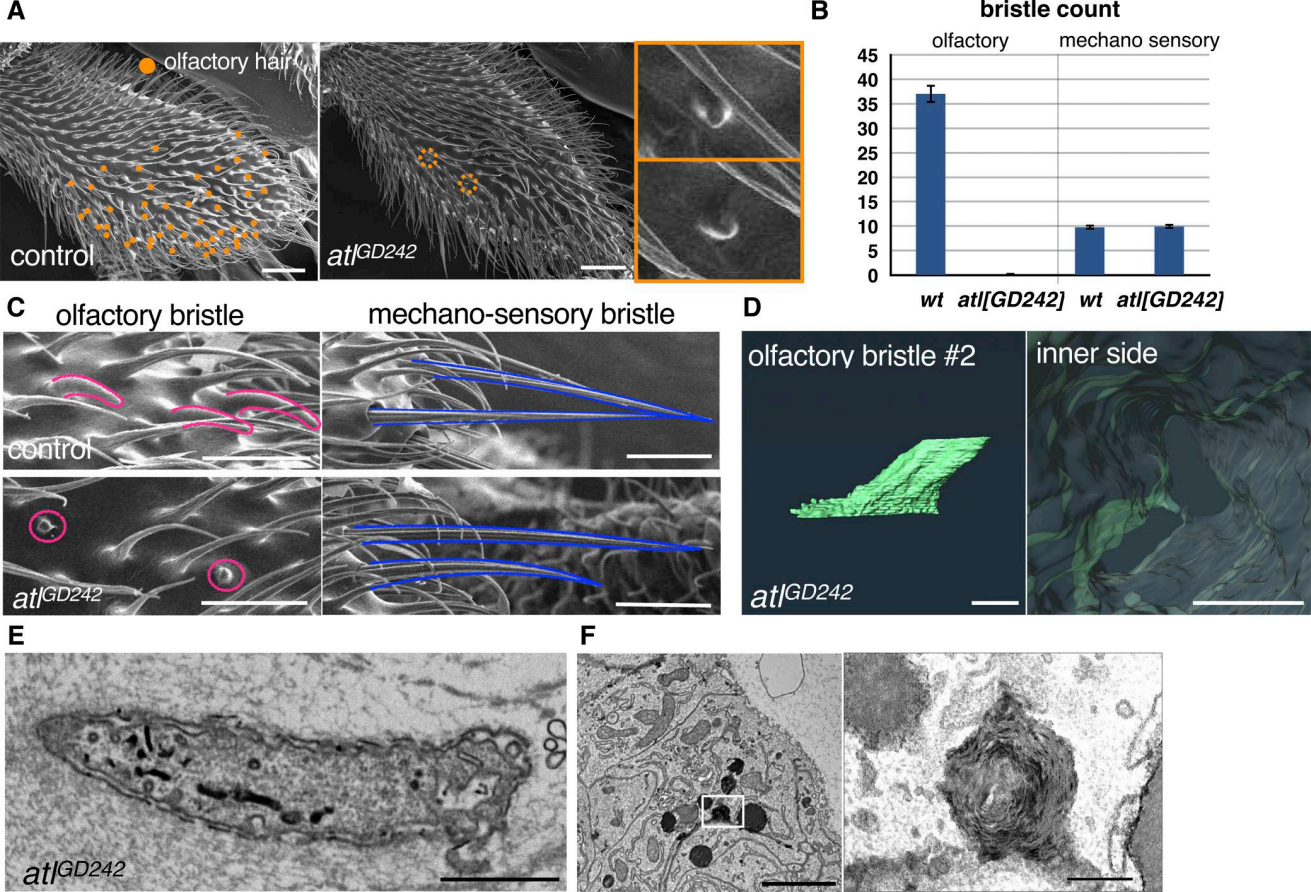
**Atl RNAi phenotype. (A)** SEM views of maxillary palp of *y w* (control) and neur > atl RNAi flies. **(B)** Count of olf and mech bristles. N of palp = 18 (WT), 16 (*atl* RNAi), and is shown as mean ± SD. No formal statistical test was applied because the data represent descriptive counts. **(C)** Enlarged views of olf and mech bristles. Note that *neur-Gal4* used in this experiment is active in both bristles. **(D)** FIB-SEM view of plasma membrane trace of *atl *RNAi olf hair cell #2. **(E)** Longitudinal TEM section of *atl* RNAi olf hair cells. Note straightened envelopes. **(F)** TEM view of condensed ER in the basal region of olf hair cell. Enlargement shows a multi-membrane “whorl” phenotype typical of stressed ER. Scale Bar: 20 µm (A), 10 µm (C), 1 µm (D), 200 nm (D inner side, approx.), 500 nm (E), 2 µm (F), and 200 nm (F enlarged).

To address the role of ER, we knocked down the ER fusion protein Atlastin (Atl) (Olso et al., 2009). This treatment caused ER fragmentation and the appearance of ER whorls (Fig. 2 E; and Fig. S3, E and F), reduced cortical Gox distribution (Fig. 2 E), the loss of PMI and PMC (Fig. 2 F, Fig. S3 D), and flattening of the envelope at 44 h APF (Fig. S3 E). In the adult maxillary palp, *atl* knockdown caused near-complete loss of olf organs, while the number of similarly treated mechanosensory bristles was unchanged (Fig. S3, A–C).

These results indicate that the ER network is required for PMC and PMI formation in the olf hair cells. Moreover, Gox-mediated ER-phagy is essential for PMI formation and maintenance of envelope curvature, itself required for nanopore formation.

### Gox recruits Ref(2)P/p62 to the plasma membrane

**Figure 3.**
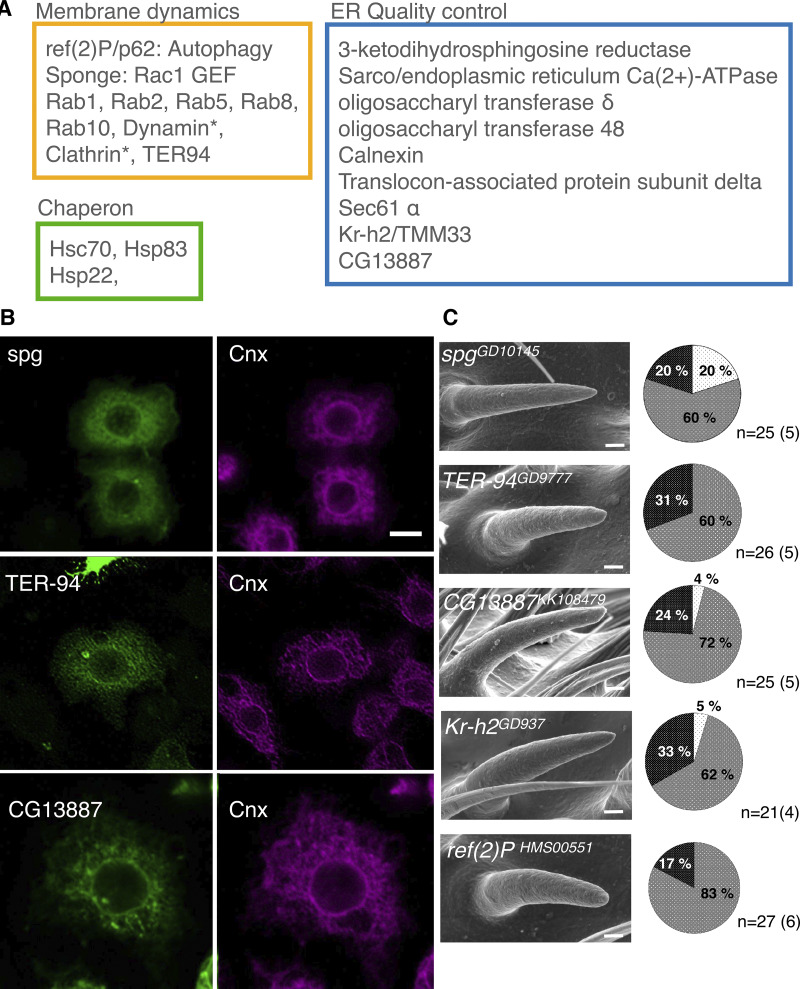
**Gox/Osi23-interacting proteins involved in nanopore formation. (A)** Mass spectrometric identification of Gox-associated proteins in S2 cells expressing Gox–Flag. An asterisk (*) indicates proteins identified from cells co-expressing Gox–Flag and Myc-Ref(2)P. The complete list of identified proteins is found in Supplementary Table S2. **(B)** ER localization of Gox-interacting proteins Spg, TER-94, and CG13887. Image of TER-94 was processed by image deconvolution. **(C)** RNAi phenotypes of Gox-interacting proteins/genes. Left: SEM image of olf bristles showing strong loss of nanopore phenotype. Right: percentage of olf bristles classified as normal (Light gray), partial (gray), and strong (black) class of nanopore loss phenotype. *n* indicates the number of scored bristles. No formal statistical test was applied because the data represent categorical frequency counts. Scale bars: 10 µm (B) and 1 µm (C).

To identify Gox effector molecules, we immunoprecipitated Gox-associated proteins from S2 cells and analyzed them by mass spectroscopy. Molecules identified included proteins involved in ER quality control (calnexin and Sec61alpha) and molecular chaperones (Hsc70), as well as molecules involved in membrane dynamics (Fig. 3 A and Table S2). Representative molecules (Sponge, Rac1 GEF; TER94, ATPase for ER membrane budding; CG13887, ER to Golgi transport) were expressed in S2 cells and were confirmed to be ER localized (Fig. 3 B). RNAi knockdown of *spg*, *TER94*, *CG13887*, *Kr-h2*, and *Ref(2)P* caused reduction of nanopores in the olf bristle (Fig. 3 C).

**Figure 4.**
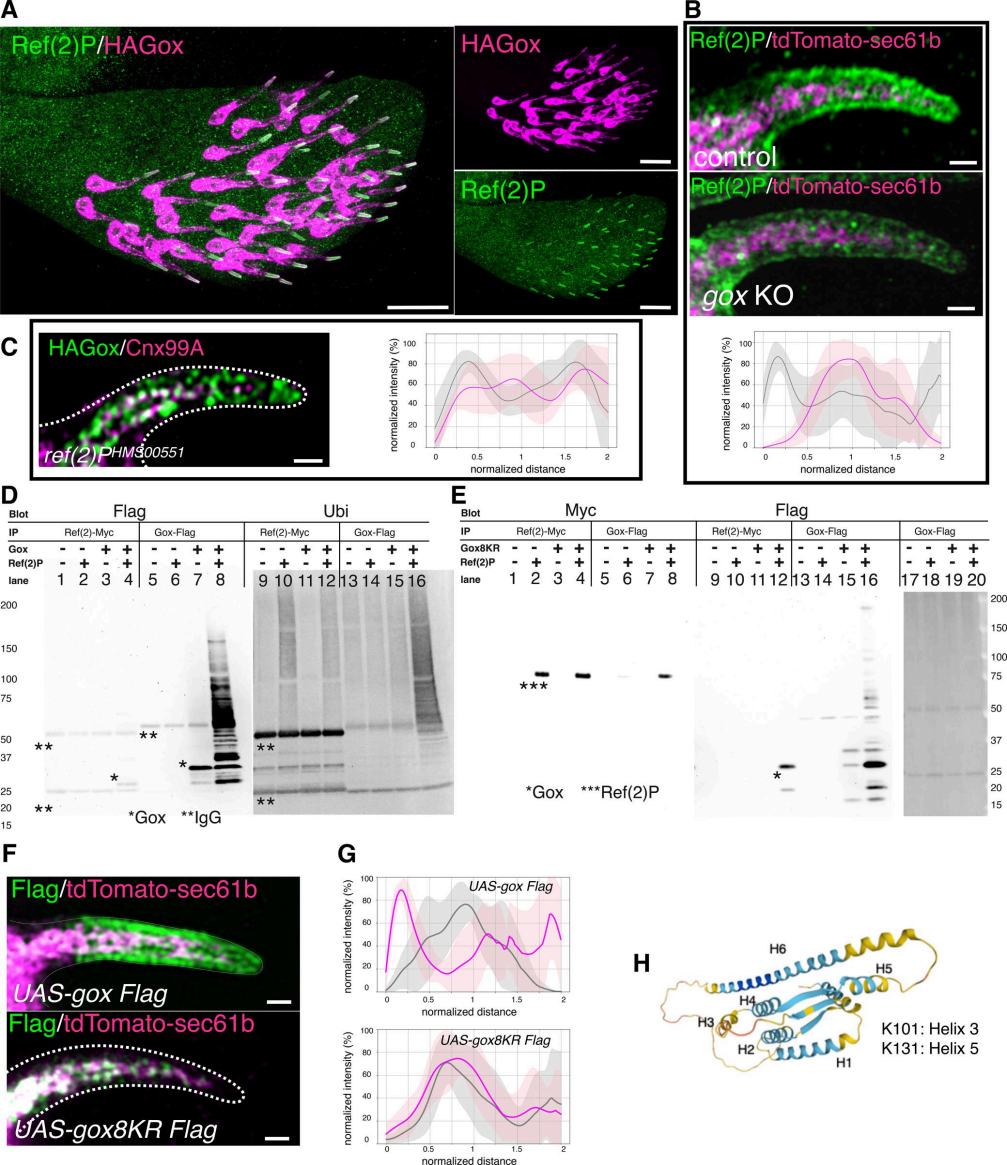
**The role of Ref(2)P in Gox localization and ubiquitination. (A)** Ref(2)P is specifically enriched in the shaft region of olf hair cells, which is labeled by HA–Gox. Maxillary palp 42 h APF. **(B)** Subcortical localization of Ref(2) in control and gox mutant olf hair cells, and their quantification. Line intensity profiles were obtained from 5–7 individual hairs per genotype using the PlotProfile function in Fiji and averaged after normalization with custom Python scripts. Gray line: Ref(2)P distribution in control; magenta line: Ref(2)P in gox KO. Error shading represents mean ± SD. **(C)** Subcortical Gox localization was lost in Ref(2)P RNAi. Right graph: quantification. Right panel: quantification of fluorescence intensity profiles. Gray line: HA–Gox in control; magenta line: HA–Gox in Ref(2)P knockdown cells. No formal statistical test was applied because the plots represent averaged fluorescence intensity distributions **(D)** Interaction of Gox2 with Ref(2)P and ubiquitination in S2 cells. Gox–Flag and Ref(2)P-Myc were co-expressed and immunoprecipitated with the tag antibodies. Immunoprecipitate of Ref(2)P containing a low molecular weight form of Gox (*, lane 4). The increased amount of high molecular weight forms of Gox was induced by Ref(2)P (compare lane 7 and 8). Note that cross-reactivity of HRP-conjugated antibodies to the IgG (marked with **) used for immunoprecipitation. **(E)** Properties of Gox8KR. Gox8KR (*) and Ref(2)P (**) were co-immunoprecipitated with each other (lane 8 and 12). *** indicates the band of Ref(2). Although Ref(2) caused a slight increase in Gox amount and molecular mass (lane 16), no specific increase in ubiquitination level was observed (lane 20). **(F)** WT gox and gox8KR were expressed by neur-Gal4. **(G)** Line intensity profiles showing the distribution of Flag (pink) and ER marker tdTomato–Sec61b (gray) in olf hair cells expressing UAS–Gox–Flag (top) or UAS–Gox8KR–Flag (bottom). Profiles were obtained and averaged as in B and C. No formal statistical test was applied; error shading represents mean ± SD. **(H)** AlphaFold 2 model of Gox/Osi23 (pLFFT score 68.12), and the location of the two mapped ubiquitinated lysine (K101 and K131) identified by the LC-MS/MS. Alpha helices are labeled (H1–H6). Molecular weight markers (kDa) are indicated for all blots, and uncropped images are provided in SourceData FS4.pdf.

We focused on Ref(2)P, a homolog of mammalian p62/SQSTM that functions as an adaptor molecule linking the autophagy molecule LC3 and protein ubiquitination (Chino and Mizushima, 2020; Nagai et al., 2021). While Ref(2)P was ubiquitously expressed as diffuse cytoplasmic puncta in most of *Drosophila* tissues (Nagai et al., 2021), it was highly enriched in the membrane-proximal region of the olf hair cell, and a subset of it overlapped with HA–Gox (Fig. 4, A and B). *gox* mutation reduced the membrane-proximal Ref(2)P (Fig. 4 B lower panel), and Ref(2)P knockdown reduced membrane-proximal Gox localization (Fig. 4 C). These results suggest that Gox and Ref(2)P interact to co-localize to the plasma membrane proximal region.

**Figure S4.**
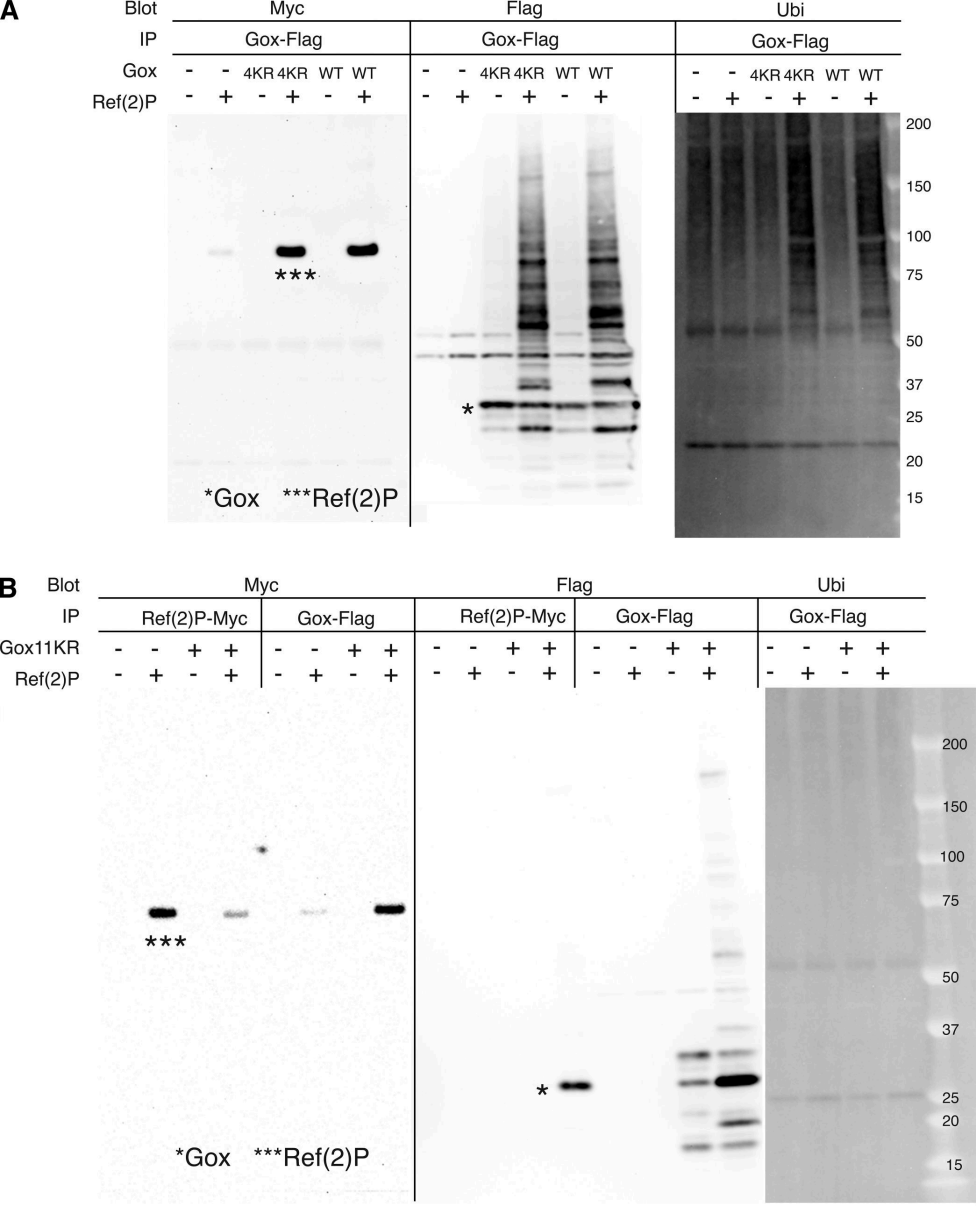
**Analysis of Gox ubiquitination. (A)** Gox4KR mutant and GoxWT were both bound to Ref(2)P and present as high molecular weight ubiquitinated forms (panel IP: Ref(2)P-Myc, blot: Flag). **(B)** Property of Gox11KR mutant. Ref(2)P associated with Gox11KR and increased its amount (compare Ref(2)P bands with and without Gox–Flag, panel IP: Gox–Flag, blot: Flag). But caused no change in ubiquitination (panel IP: Gox–Flag, blot: Ubi). The Gox11KR phenotype was identical to that of Gox8KR. The bands of native Gox and Ref(2)P are indicated by asterisks (*, ***). Source data are available for this figure: SourceData FS4.

Gox expressed in cultured S2 cells was detected as multiple bands of relative molecular mass of 25–30 kd (Fig. 4 D, lane 7). Co-expression of Ref(2)P increased the amount of Gox and its molecular size up to 150 kd that reacted with an anti-polyubiquitin antibody (Fig. 4 D, lane 8 and 16). Although mammalian p62 is known to bind ubiquitinated proteins, Gox bound to Ref(2)P was exclusively the native form of 28.6 kd with no ubiquitin (Fig. 4 D, lane 4). We identified two ubiquitination sites, K101 and K131, by mass spectrometry (Fig. 4 H and Table S4). Substitution of the last eight, or all 11, lysines of Gox to arginine (Gox8KR, Gox11KR, including K101 and K131), eliminated ubiquitination but preserved its binding to Ref(2)P (Fig. 4 E and Fig. S4 B). Substitution of the last four lysines, leaving K101 and K131 intact (Gox4KR), retained the WT level of binding function to Ref(2)P and ability to be ubiquitinated (Fig. S4 A).

To test the function of Gox ubiquitination, Gox8KR–Flag and its WT form (Gox–Flag) were expressed in the olf hair cell. While Gox–Flag was found in both ER and subcortical region, Gox8KR–Flag was found only in the ER (Fig. 4, F and G). Based on those results, the following scenario was deduced. In the olf hair cell, Gox in the ER membrane associates with Ref(2)P, and this interaction triggers ubiquitination of Gox and subsequent dissociation of Ref(2)P. The part of ER enriched with ubiquitinated Gox is excised and translocated to the plasma membrane. Ubiquitination-deficient Gox8KR–Flag failed to exit from the ER.

### PMI is the site of ER–membrane interaction

**Figure 5.**
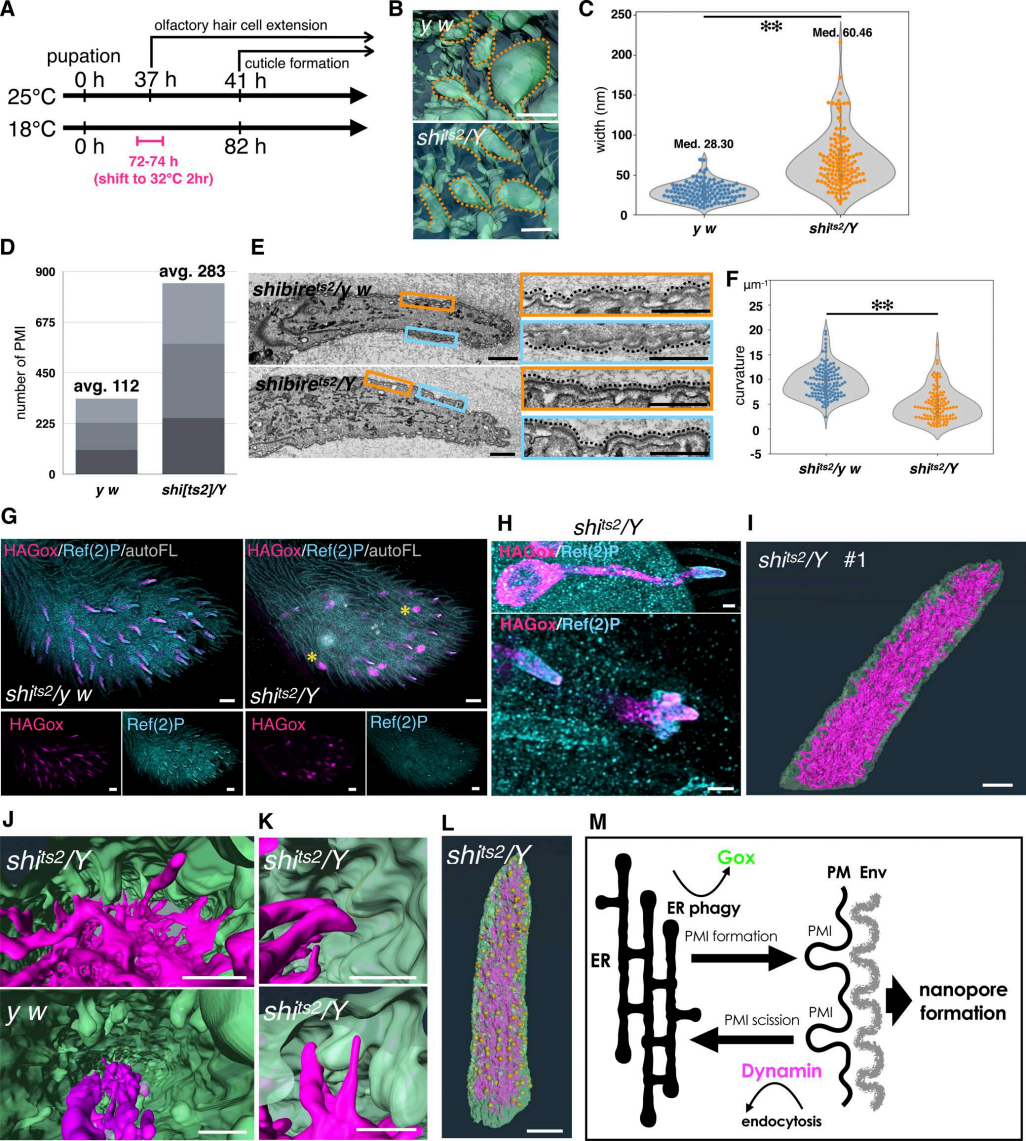
**Dynamin–Gox interaction. (A)** Temperature-shift protocol for inactivating dynamin in *shi*^*ts2*^ mutants. **(B)** PMI in control and *shi^ts2^* mutants olf hair cells reconstructed by FIB-SEM. **(C)** PMI neck width measured from three independently reconstructed olf hair cells per genotype. Data were plotted as violin plots showing the distribution and median values. (*shi*^*ts2*^*/y w*: 118 points from three individual olfs; *shi*^*ts2*^*/Y*: 131 points from three individual olfs). Statistical significance was determined using the Mann–Whitney U test (two-sided), **: P < 0.001. **(D)** Number of PMIs quantified from FIB-SEM reconstructions of three olf hair cells per genotype. Counts were made from three independently reconstructed olf hair cells for each genotype (*y w* and s*hi*^*ts2*^*/Y*). No formal statistical test was applied because the data represent simple counts. **(E)** TEM views of *shi*^*ts2*^*/y w* (control) and *shi*^*ts2*^*/Y* (mutant). **(F)** Quantification of envelope curvature. Point curvatures were calculated using the Kappa plugin in Fiji, and the median curvature of each envelope fragment was used as a representative value. Data were compared using the Mann–Whitney U test (two-sided). **: P < 0.01. **(G)** Maxillary palp of *shi*^*ts2*^*/y w* (control) and *shi*^*ts2*^*/Y* (mutant) stained for expression of HA–Gox and Ref(2)P. Asterisk indicates completely internalized olf hair cells. **(H)** Enlarged views of *shi*^*ts2*^*/Y* olf hair cells, showing normal (top and lower-left) and partially invaginated (lower right) phenotypes. **(I)** FIB-SEM view of plasma membrane and ER of *shi*^*ts2*^*/Y* olf #1. **(J)** Relationship of ER and plasma membrane in *shi^ts2/^Y* and *y w* (control). **(K)** ER–plasma membrane contacts in *shi*^*ts2*^*/Y*. **(L)** Distribution of ER–plasma membrane contact site (yellow dot) in *shi*^*ts2*^*/Y* olf #1. **(M)** Model of plasma membrane and ER interaction underlying nanopore formation. PMI formation is sustained by the stimulation by Gox and the clearance by dynamin. This dynamic interaction is coupled to ER-phagy, which supplies excessive lipids to the plasma membrane that buckles under the confinement by apical ECM. Envelope formation follows plasma membrane curvature. Bar: 100 nm (approx.), 1 µm (E, left), 200 nm (E, right), 10 µm (G), 1 µm (H, I), 200 nm (J, approx.), 100 nm (K, approx.), and 1 µm (L, approx.).

**Figure S5.**
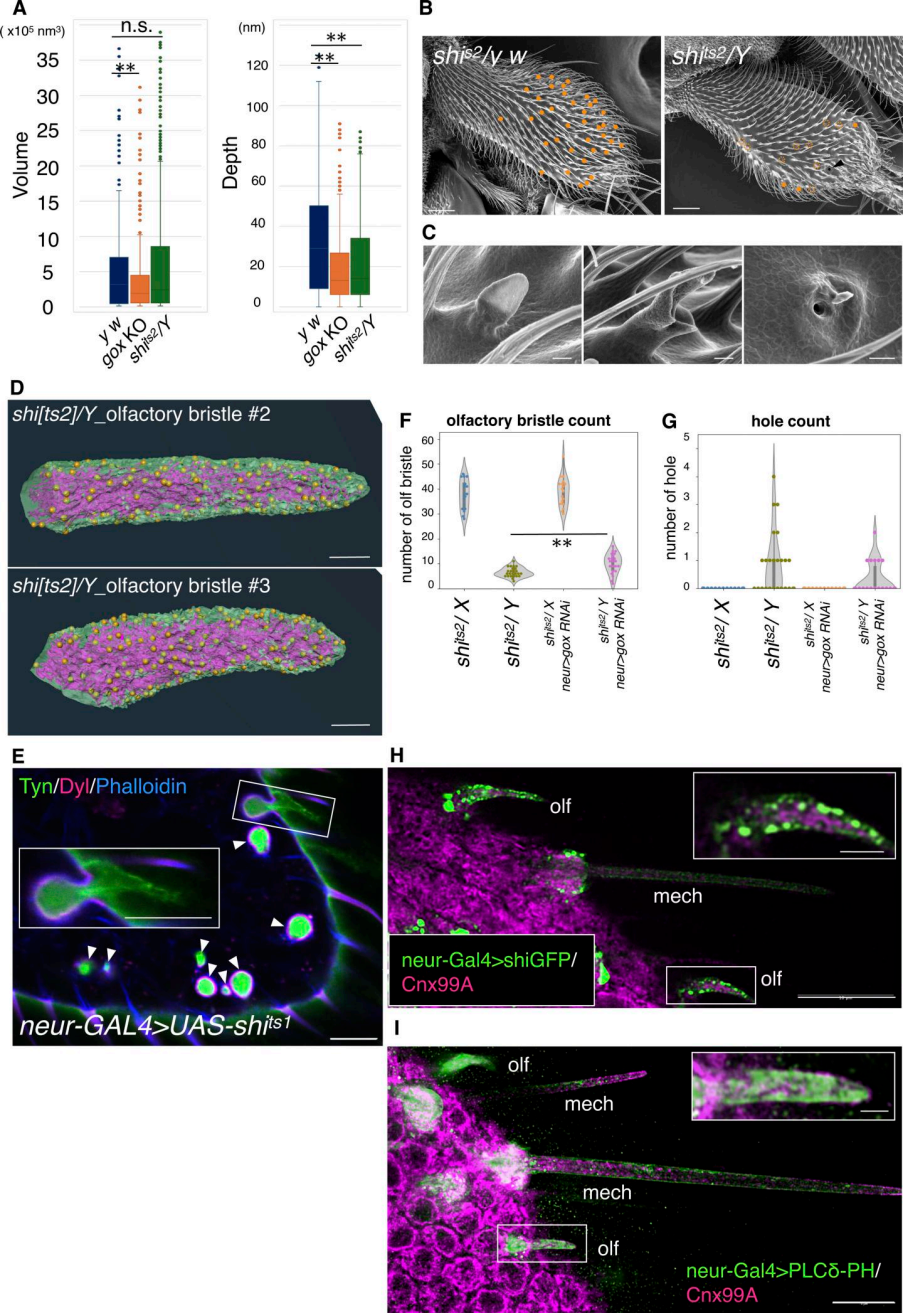
**Analysis of dynamin-deficient olf hair cells. (A)** Quantification of PMI volume and depth measured from FIB-SEM reconstructions of three olf hair cells of *y w*, *gox*, and *shi*^*ts2*^*/Y* pupae. Data are shown as box-and-whisker plots indicating the median, interquartile range, and full distribution. Statistical significance was determined using the Mann–Whitney U test (two-sided). **: *P* < 0.01; n.s.: not significant. **(B)** Phenotype of maxillary palp bristles. Female (*shi*^*ts2*^*/X*, control) and male (*shi*^*ts2*^*/Y*, experimental) progenies from the same batch of heat-treated flies are compared. Filled dot indicate olf bristles of normal length and abnormal morphology, respectively. Arrowhead in the *shi*^*ts2*^*/Y* panel indicates the open hole of the cuticle. **(C)** Enlargement of abnormal olf bristles of *shi*^*ts2*^*/Y*. Left to right: short, branched, and hole phenotype. **(D)** FIB-SEM views of olf hair cells #2 and #3 of *shi*^*ts2*^*/Y* showing ER, plasma membrane, and their contact site (yellow dot). **(E)** Maxillary palp expressing *UAS-shi*^*ts1*^ driven by the *neur-Gal4* driver. Apical ECM marker: mVenus-Tyn and mScarlet-Dyl. Arrowhead indicates the lumen of invaginated olf cells containing secreted mVenus-Tyn and surrounded by mScarlet-Dyl. Enlargement shows a hair cell–like remnant of Tyn left outside of the invaginated olf cell. **(F)** Number of olf hairs on the maxillary palp of *shi*^*ts2*^ mutants and *gox* knockdown combinations. **(G)** Number of pores of maxillary palp in the same genotypes. Each data point represents one maxillary palp (*n* = 17 for *shi^ts2^*/*X*; *n* = 25 for *shi*^*ts2*^/*Y*; *n* = 18 for *shi*^*ts2*^/*X*; *gox* KD; *n* = 22 for *shi*^*ts2*^/*Y*; *gox* KD). Data are shown as mean ± SD, and statistical significance was determined using the Mann–Whitney U test (two-sided). **: *P* < 0.01; n.s.: not significant. Note that *neur > gox RNAi* partially suppressed the *sh*^*ts2*^*/Y* phenotype. **(H)** shi-GFP expression driven by the *neur-Gal4* driver in the olf and mech at 42 h APF. GFP signal is highly enriched in the cortical region of the olf hair cell, but its intensity is much lower in the mech. Socket cells of olf and mech accumulate about the same levels of GFP signal. **(I)** PLCdelta-PH-EGFP, a marker for PI(4,5)P₂, driven by the *neur-Gal4* driver. EGFP signal strongly labels the cortical region of the olf hair cell and weakly that of the mech hair cell. Bar: 20 µm (B), 1 µm (C) 1 µm (D), and 10 µm (E, H, and I). Asterisk: **, P < 0.001 (A and F); (G). Statistical significance was determined by Mann–Whitney U test.

Next, we studied the interaction of Gox-containing ER-derived vesicles and the plasma membrane. The PMI resembles the clathrin-coated pit that forms during endocytosis, a rapid process completed in ∼90 s by dynamin-catalyzed scission (Loerke et al., 2009). To capture the interaction of PMI and ER in the snapshot images of EM, the PMI scission reaction was arrested by reducing the activity of dynamin using the *shibire*^*ts2*^ (*shi*^*ts2*^) mutation (Kosaka and Ikeda, 1983). A temperature shift was applied at the time equivalent of APF 36–37 h at 25°C, following the protocol optimized to maximize the effect on olf hair formation while avoiding developmental arrest (Fig. 5 A). This transient inactivation of dynamin caused ∼2.5-fold increase in the number of PMI (Fig. 5, B and D). Measurement of the 3D views of control and fully elongated *shi*^*ts2*^ olf hair cells at 44 h APF (at 25°C) revealed the increase in the neck width (Fig. 5 C), volume, and depth of PMI (Fig. S5 A). TEM views showed a reduction of envelope curvature in elongated olf hair cells (Fig. 5, E and F). The cortical localization of HA–Gox and Ref(2)P was comparable with the control (Fig. 5, G and H).

In the *shi*^*ts2*^ olf hair cell, ER was markedly overexpanded, and numerous tubular ERs were extended toward the plasma membrane (Fig. 5 I). In magnified views, frequent association of PMI and PMC with extended ER was observed (Fig. 5, J and K). The results demonstrated that PMI is undergoing frequent dynamin-driven scission, and this process is dependent on Gox. *shi*^*ts2*^ caused the arrest of PMI scission and ER-phagy. The ER–plasma membrane contact sites mapped on the surface of *shi*^*ts2*^ olf hair cells showed a scattered appearance resembling the nanopore pattern in WT olf bristles (Fig. 5 L and Fig. S5 D). These data revealed an unexpected interaction of endocytosis and ER-phagy, coupled by Gox and dynamin, suggesting a mechanistic link between ER and PMI.

### Dynamin mediates the plasma membrane control of ER-phagy

The emerged flies of *shi*^*ts2*^ showed a significant reduction in olf bristles (Fig. S5, B and F), whereas the numbers and shapes of mech bristles and spinules were unaffected. High-magnification imaging of the olf bristles showed variable phenotypes of full elongation, malformation, and complete loss, leaving a hole in the cuticle (Fig. S5 C). At APF 44 h, olf hair cells showed various morphologies, including normal elongation, cup-shaped indentation, and a total internalization subjacent to the epidermis (Fig. 5 G asterisk, Fig. 5 H). Olf hair cells expressing the dominant-negative *shi*^*ts1*^ construct showed a similar internalized phenotype (Fig. S5 E). By labeling the apical ECM marker Tyn, we visualized the cavity formed by the internalized cells filled with ECM (Fig. S5 E, arrowhead) and hair cell–shaped ECM remnants associated with totally invaginated olf hair cells (Fig. S5 E, inset), suggesting that dynamin-deficient olf hair cells elongated and then retracted inward at 44 h APF, leaving emptied apical ECM outside.

Dynamin was found among the Gox-associated proteins (Fig. 3 A). Quantitative analysis of FIB-SEM images revealed that *gox* and *shi*^*ts2*^ showed opposite phenotypes in the PMI volume (Fig. S5 A). We next asked whether the two genes genetically interact. The combination of gox-RNAi to *shi*^*ts2*^ caused significant suppression of the loss-of-olf and epidermal hole phenotypes of *shi*^*ts2*^ in the adult (Fig. S5, F and G). shi-GFP expressed by the neur-Gal4 driver was highly accumulated in the olf hair cells but not in the mechanosensory hair cells (Fig. S5 H). A high-magnification view showed that shi-GFP was associated preferentially with the plasma membrane, but not with the ER (Fig. S5 H, inset). The olf hair cells also accumulate high levels of PLCdelta-PH-EGFP, a marker for PI(4,5)P_2_ (Stauffer et al., 1998; Várnai and Balla, 1998). Since dynamin/shibire is known to bind PI(4,5)P_2_ with its PH domain (Vallis et al., 1999; Achiriloaie et al., 1999), the high enrichment of PI(4,5)P_2_ in the olf hair cells explains the plasma membrane enrichment of shi-GFP in this cell type. The results suggest that dynamin associated with the plasma membrane sustains the autophagic degradation of ER and the maintenance of the olf hair cell protrusion.

## Discussion

### The role of Gox in the formation of curved envelop and nanopore

Cuticles are secreted from the plasma membrane in different forms. Flat envelopes are produced from membrane protrusions of epidermal cells (plasma membrane plaque [Locke and Huie, 1979]) and from flat membranes rich in subcortical actin filaments (mechanosensory bristle [Tilney et al., 1995], spinule, this study). The olf hair cells are unique in that they produce a wavy envelope that later forms nanopores. This cell type also uniquely develops an extensive ER network, and its plasma membrane is convoluted (PMC) and occasionally invaginated (PMI), suggesting that the plasma membrane area is overexpanded relative to the volume of the olf hair cells. When the ER network is disrupted by *atl* RNAi, PMI and PMC are lost, and the envelope curvature fails to appear. Therefore, the ER network is required for shaping PMC and PMI of the olf hair cells, likely by serving as a source of plasma membrane lipid.

*gox* specifically affects PMI and the maintenance of envelope curvature. Our results suggest two-step regulation of envelope curvature by ER. The initial curvature at 44 h APF is induced by the full activity of the tubular ER network, possibly the envelope following the convoluted plasma membrane (step 1). Gox maintains this curvature by promoting PMI and ER interaction, and PMI helps to maintain and further develop the curved envelope (step 2). The ER network contributes to step 1 by providing excess membrane to form PMC. Gox contributes to step 2 by promoting PMI formation to maintain the envelope curvature.

Gox is present in the ER membrane of the olf hair cell. Plasma membrane of the shaft part of the olf hair cell is exposed to the outside of the tissue, where a fraction of ER is converted into the membrane-associated type II vesicles that are further changed to type III vesicles with autophagosome-like morphology. This ER conversion is triggered by the association of Gox with Ref(2)P and its subsequent ubiquitination. Ubiquitination of Gox may contribute to a change in ER membrane curvature and scission by increasing the molecular mass of its cytoplasmic part, as reported for the role of ubiquitination of the ER membrane protein FAM134B (Foronda et al., 2023; González et al., 2023; Jiang et al., 2020).

The mode of Ref(2)P recruitment to Gox is clearly different from the known function of its mammalian counterpart, p62/SQSTM1, which recognizes ubiquitinated proteins under stressed conditions and recruits LC3 for autophagy of organelles (Chino and Mizushima, 2020; Anding and Baehrecke, 2017; Huang et al., 2025). On the other hand, cortical condensation of Ref(2)P under the non-stressed condition was observed only in the shaft part of the olf hair. Ref(2)P binds non-ubiquitinated Gox, and ubiquitination of Gox occurs as a consequence of its interaction with Ref(2)P. This unique interaction may direct the Gox-associated ER to the plasma membrane to serve as a source of lipids transferred to other membrane compartments. It is known that the autophagosome is converted into the lamellar body to serve as a lipid reservoir for extracellular delivery to coat the internal surface of the air-filled organs of animals (swim bladder of zebrafish and lung of the mouse, Morishita et al., 2020). Alternatively, lipids may be supplied directly from ER-derived vesicles to the plasma membrane by lipid exchangers (Ma et al., 2010; Giordano et al., 2013; Chung et al., 2015; Nakatsu and Kawasaki, 2021) or lipid transfer channels (Levine, 2022; Neuman et al., 2022).

### Bidirectional interaction of the ER and plasma membrane sustains ER-phagy

The ER and plasma membrane (PMI) contact is the intersection point of two pathways. From the ER, Gox-Ref(2) interaction triggers scission of the ER and its access to the plasma membrane. From the plasma membrane, dynamin promotes degradation of the ER. Inhibition of dynamin simultaneously arrested endocytosis and ER-phagy, indicating that both Gox in the ER and dynamin on the plasma membrane cooperatively promote ER-phagy. The two processes, ER-phagy and PMI formation, requiring Gox, are functionally coupled at PMI. This coupling allows the ER to access the plasma membrane, enabling the transfer of lipids to expand the plasma membrane surface that convolutes in the limited space covered by the apical ECM (Itakura et al., 2026, Science Advances in press). Excessive plasma membrane growth under the confined environment would cause the plasma membrane to buckle. The space between the envelope and plasma membrane is filled with apical ECM that includes the zona pellucida–like domain protein Dusky-like (Itakura et al., 2026, Science Advances in press). The newly forming envelope would follow the membrane curvature to become wavy (Fig. 5 M). The ER may also provide lipids and secreted materials through PMI to the prospective pore region of the curved envelope, which is distinctly structured and detergent sensitive (Ando et al., 2019).

Dynamin was identified as a microtubule-associated protein (Shpetner and Vallee, 1989) and is required for proper microtubule distribution (Gonzalez-Bellido et al., 2009). Microtubule bundles may apply a pulling force to PMI by coupling with dynamin, transmitting the force to the envelope via apical ECM to promote envelope curving. While the PMI-microtubule mechanical coupling is transient, limited by the lifetime of PMI, dynamin inhibition in *shi*^*ts2*^ mutants allows longer coupling to cause mechanical instability of the olf hair cell to buckle internally.

### Limitations and concluding remarks

This work highlighted PMI as the focal point of ER–plasma membrane interaction. We noted numerous ER–PMI contacts in dynamin-arrested olf hair cells. In our limited example of FIB-SEM images of control olf hair cells (*N* = 3), ER–PMI contact is very rare. This is probably because ER–PMI contact is a transient event that occurs over a short period during the rapid process of endocytosis. Large-scale FIB-SEM analysis would be required to capture the critical moment of ER–PMI contact. An alternative approach would be to observe the event with high-speed super-resolution imaging. However, the nanoscale size of the contacts, short contact duration, and the location of the olf hair cells deep under the pupal cuticle make this experiment a challenging one. Mechanisms of how the ER-resident Gox promotes PMI formation, and how dynamin, which is associated with PMI, sustains ER-phagy, are not understood at this moment. Addressing those questions would require identifying the molecules that mediate the contact between the ER and the plasma membrane (Saheki and De Camilli, 2017).

The similarity of the nanopore distribution and the pattern of ER–plasma membrane contact in *shi*^*ts2*^ olf hair cells supports a model in which the ER network serves as a template for nanopore patterning, as previously suggested in butterfly scale cells, where complex invagination of the plasma membrane associated with ER precedes the formation of cuticular photonic nanocrystals (Ghiradella, 1974, 1989, 1991; Saranathan et al., 2010). The template function of ER in specifying plasmodesmata, the nanoscale (50 nm diameter) intercellular channel across the plant cell wall, is documented (Lucas and Lee, 2004; Nicolas et al., 2017). Gox/Osi23, an insect-specific innovation, enabled the ER to acquire its novel function in cuticle patterning by permitting the ER–PMI interaction that is coupled to the autophagy pathway. Future investigation into the roles of *Osi* genes in other nanostructures in *Drosophila* (e.g., corneal nipples and tip pores) and structurally colored scales in butterflies and beetles will open up rich new possibilities in the manipulation and design of insect cuticles with novel or modified surface functions.

## Materials and methods

### Key resource table

Table S1 lists reagents (antibodies, chemicals, equipment, fly strains, plasmids, and software used in this study).

### Oligonucleotide primer list

Table S3 lists primer sequences used in this study.

### Fly stocks and husbandry

The flies were maintained on standard cornmeal-yeast food at 25°C unless otherwise noted. Fly pupae at the white prepupal stage were picked up and staged.

### Pupal tissue preparation

For fluorescent microscopy, olf hair cells of maxillary palp were analyzed. For FIB-SEM, TEM, and APEX2 analyses, the dorsal-medial region of the An3 rich in olf organs was chosen for analysis. Gox/Osi23 is expressed in both maxillary palp and An3 and is required for olf nanopore formation (Ando et al., 2019; Sun et al., 2024).

### Temperature shift experiment

The temporal inactivation of dynamin using a *shi*^*ts2*^ mutation was designed according to the developmental time course at different temperatures (Ashburner et al., 2005). Virgin females of *shi*^*ts2*^ were crossed to *y*^*1*^*w*^*1118*^ males, and the progenies were cultured at 18°C before staging at the white prepupal stage. Pupae at 72 h APF (equivalent to 36 h APF at 25°C) were heat treated at 32°C for 2 h in 1.7-ml microfuge tube placed in a block incubator and then returned to 18°C until eclosion. Males (*shi*^*ts2*^*/Y*, hemizygote mutants) and females (*shi*^*ts2*^/*X*, heterozygote control) were analyzed by field emission SEM (FE-SEM). For the genetic interaction assay with *gox* RNAi, male *gox*^*kk103932*^; *neur-GAL4/TM6c SbDfd-GMR-YFP* strain was crossed to virgin *shi*^*ts2*^ females.

### APEX2 tagged–gox knock-in vector construction

APEX2 was targeted to the C terminus of the Gox signal peptide so that APEX2 is retained at the N terminus of the mature Gox protein after signal peptide removal. Oligonucleotides used in this experiment are listed in Table S3. The knock-in strain with APEX2 tagged–*gox* was constructed by following the scarless gene-editing protocol from the laboratory of Kate O'Connor-Giles (Gratz et al., 2014) (https://flycrispr.org/scarless-gene-editing/). To build the donor plasmid, 1 kb each of the 5′ and-3′ homology arms flanking the target site were amplified using KOD One (Toyobo) from the *gox* genomic rescue construct (Ando et al., 2019). The APEX2 sequence amplified from pAPEX2-Dyl and the 3xP3-DsRed cassette with PBac transposon ends were amplified. The sequence of gRNA target sites in the homology arms was mutated using the primer set SI7–SI8 and SI9–SI10 to prevent cleavage of the donor vector. The four fragments were ligated using the In-Fusion HD Cloning kit (Takara) into the pBS SKII vector cut with EcoRI (Takara). The plasmid sequence was confirmed by DNA sequencing. Oligonucleotide primers used in this procedure are listed in Table S3.

Three gRNAs to the sequences around 300 bps upstream and downstream of the target site were designed https://targetfinder.flycrispr.neuro.brown.eduhttps://www.flyrnai.org/evaluateCrispr/input and were inserted into the BbsI-HF site of pCFD3-dU6 plasmid (#49410; Addgene).

### Genome editing

A mixture of the three gRNA vector plasmids (50 ng/μl each) and the donor plasmid (150 ng/μl) was injected into the *y*^*2*^*cho*^*2*^*v1 P{nos-Cas9, y+, v+}1A/CyO* strain. Transformed flies were identified based on the red fluorescence in the eye, and balanced strains were established. Those flies were crossed with *w*^*1118*^*; CyO, P{Tub-PBac/T}2/wg*^*Sp-1*^*; l(3)/TM6B, Tb1* to excise the 3xP3-DsRed element, and fluorescence-negative individuals were selected. Correctly edited strains were identified by PCR and DNA sequencing.

### Detection of APEX2 by electron microscopy

44–45 h APF pupae were removed from the pupal case and cut open in the abdomen and were transferred to fixation buffer (2.5% glutaraldehyde, 2% formaldehyde, and 0.1 M sodium cacodylate buffer, pH 7.4) on ice and were stored at 4°C overnight. The tissues were washed three times for 10 min each in 0.1 M cacodylate buffer. Pupal cuticles and the body were removed from the head tissues and were washed with 0.1 M cacodylate buffer three times for 10 min.

DAB treatment and silver-gold enhancement were performed using a modification of the published method (Rae et al., 2021). Fixed heads were incubated in 200 mM glycine in 0.1 M cacodylate buffer for 5 min on ice and rinsed with 0.1 M cacodylate buffer for 10 min. DAB solution was prepared by mixing 2 mg/ml DAB in H_2_O (prepared from DAB tablet, Sigma-Aldrich) with an equal volume of 0.1 M sodium cacodylate buffer on ice and filtering through a 0.22-μm syringe filter. Fly heads were incubated in 500 μl of DAB solution on ice for 30 min. The solution was replaced with DAB solution containing 10 mM H_2_O_2_, and incubation was continued on ice for 30 min to 1 h. After the DAB reaction, samples were washed three times with 0.1 M sodium cacodylate for 10 min, with H_2_O four times for 15 min each, and with 1% BSA in 20 mM glycine for 20 min, on ice. Next, the samples were incubated in 1% BSA and 20 mM glycine for 20 min at room temperature and prewarmed at 60°C for 10 min, followed by incubation in prewarmed silver enhancement solution (20:1:2 mixture of 3% hexamethylenetetramine, 5% silver nitrate, and 2.5% disodium tetraborate) for 15 min at 60°C for 15 min. Samples were washed with H_2_O three times for 5 min at room temperature, incubated with 0.05% tetrachlorogold (III) acid trihydrate in H_2_O for 5 min at room temperature, washed in H_2_O, and incubated with 2.5% sodium thiosulphate for 4 min at room temperature. Samples were washed with H_2_O, incubated with 2.5% sodium thiosulphate for 4 min at room temperature, and then washed with H_2_O three times. The samples were post-fixed with 1% osmium tetroxide and 1% (wt/vol) potassium ferrocyanide in H_2_O. After washing three times for 5 min each in H_2_O, samples were immersed in 1% uranyl acetate at 4°C overnight. The stained tissues were washed three times for 5 min each in water, and the tissues were subsequently dehydrated in a graded ethanol series. The following embedding and sectioning procedures were the same as for the FIB-SEM and the TEM procedure described below.

### 3D cell imaging by FIB-SEM

Pupal head fixation followed the procedure for APEX2 staining up to the removal of pupal cuticles. Samples were washed three times for 10 min each in 0.1 M cacodylate buffer and post-fixed in 2% OsO_4_, 1.5% (wt/vol) potassium ferrocyanide, and 2 mM CaCl_2_ in 0.15 M sodium cacodylate buffer (pH 7.4) for 2 h on ice in the dark. The fixed tissues were then washed three times for 10 min each in water and immersed in 1% thiocarbohydrazide for 1 h at 60°C. The tissues were then washed five times for 5 min each in water at room temperature and fixed again in 2% OsO_4_ for 1 h on ice. The fixed tissues were washed three times for 5 min each in water and stained *en bloc* in 1% uranyl acetate in H_2_O overnight at 4°C. The stained tissues were washed three times for 5 min each in water, and the tissue was stained with lead aspartate solution at 60°C for 1 h. The tissues were washed three times for 5 min each in water at room temperature, dehydrated in a graded ethanol series (30, 50, 70, 80, 85, 95, and 99.5%) for 5 min each on ice, and transferred to 100% ethanol for 15 min on ice. After dehydration, the tissues were incubated in ice-cold 100% acetone for 5 min and incubated for 5 min with 100% acetone at room temperature. For resin substitution, samples were immersed in a 3:1 mixture of 100% acetone and resin (8.8 g Epon812, 2.7 g DDSA, and 0.23 ml DMP-30) at room temperature overnight, in a 1:1 mixture of acetone/resin for 6 h, and in 1:3 acetone/resin overnight. Then samples were incubated in 100% resin at room temperature overnight. The resin was replaced by a fresh one and was polymerized by sequential incubation at 60°C for 12 h, 45°C 12 h, 65°C 48 h, and 70°C 24 h.

After complete polymerization, the excess resin was trimmed, and the tissue block was mounted on an aluminum pin with electrically conductive glue, with the back side of the head on the top. Using an ultramicrotome (EM UC7 microscope; Leica), the block was cut from the back to front direction until the antennae were exposed and coated with osmium (Tennant 20; Meiwafosis). Serial FE-SEM imaging of the block surface cut with a focused ion beam was performed (Helios G4 UC and Aquilos2, Thermo Fischer Scientific). Typically, 4 × 4 × 10-nm voxel (x-y-z) images (5000×, ∼1000 sections, 1.5 kV, 0.1 nA) of 10 × 20 µm area were obtained from the inside to the outside direction at the medial-dorsal region of the An3 rich in sensilla basiconica.

### Data segmentation and analysis

Automatic alignment of image stacks and signal normalization between slices, 3D reconstruction, and manual segmentation of the image stacks were performed using the Amira software Version 2020.2 (Thermo Fisher Scientific).

### TEM

Sample fixation and embedding were performed according to the protocol of FIB-SEM sample preparation. After trimming the embedded tissue, ultrathin sections (∼50 nm) were cut and mounted on 200-mesh copper grids. Specimens were observed using a JEM-1400Plus transmission electron microscope (JEOL) at 100 kV accelerating voltage.

### FE-SEM

The adult heads were dissected in PBS and then rinsed with 0.1 M cacodylate buffer three times for 5 min each and incubated in fixation buffer 1 (2% paraformaldehyde, 2.5% glutaraldehyde, and 0.1 M cacodylate buffer) at 4°C overnight. The samples were rinsed with 0.1 M cacodylate buffer three times at room temperature for 5 min each. Then, the samples were incubated in fixation buffer 2 (1% OsO_4_ and 0.1 M cacodylate buffer) on ice for 120 min in a light-shielded condition. Samples were further rinsed in water three times on ice with the light-shielded condition and subsequently dehydrated in a gradient of ethanol concentration, from 25 %, 50%, 75 %, 80 %, 90 %, 95 %, 99.5 %, and 100% for 10 min each at room temperature. The final 100% ethanol was dehydrated by the addition of a molecular sieve (NACALAI TESQUE). The samples were dried by storing them in the desiccator for 3–4 days. After dehydration, the heads were mounted on double-sided carbon tape on a brass pedestal and coated with OsO_4_ at ∼15 nm thickness using an osmium coater (Tennant 20, Meiwafosis Co., Ltd.). The samples were observed with the field emission scanning electron microscope (JSM-IT700HR; JEOL).

### Fluorescence microscopy

Super-resolution images of the olf sensilla and spinules on the maxillary palp were acquired by the confocal microscopes equipped with Plan-Apochromat 63×/1.4 NA oil immersion objective lenses and the Airyscan detectors (LSM 880 and LSM980; Zeiss). Images were reconstructed by Airyscan processing with the 3D or 2D auto setting of the Zen software (Carl Zeiss) and analyzed with ImageJ/Fiji (Schindelin et al., 2012; Schneider et al., 2012).

For sample preparation, 44 h APF pupae were dissected out from the pupal case and fixed with 4% paraformaldehyde and 0.3% Triton X-100 in PBS overnight at 4°C. Pupal cuticles were removed from the head with sharp forceps (Inox #5). Pupal heads were washed three times for 10 min with PBST (0.3% Triton X-100 in PBS) at room temperature. Samples were blocked with 0.3% Triton X-100 and 0.1% BSA in PBS (PBSBT) for 20 min at room temperature and incubated with first antibody solution in PBSBT overnight at 4°C. Samples were washed three times with PBST and once with PBSBT, each for 10 min, and incubated with second antibody in PBSBT at room temperature for 2 h. After washing three times for 10 min with PBST, the heads were incubated in the mounting solution (SlowFade Diamond Antifade Mountant, S36972; Thermo Fisher Scientific). The maxillary palp was detached from the head and mounted on a glass slide.

The following antibodies and staining reagents were used (Table S1): rat anti-HA (1:500; Roche, 3F10), mouse anti-Calnexin99A (1:10, DSHB, Cnx99A 6-2-1), rabbit-anti RFP (1:200, BD Bioscience, and 1:200, MBL), and rabbit anti-Ref(2)P (1:1000, gift from Tamaki Yano at Tohoku University, Sendai, Japan). The following secondary goat antibodies were used at 1:200: anti-rabbit Alexa 488 highly cross-adsorbed (A11034; Thermo Fisher Scientific), anti-rabbit Alexa 555 cross-adsorbed (A21429; Thermo Fisher Scientific), anti-mouse Alexa 488 highly cross-adsorbed (A11029; Thermo Fisher Scientific), anti-mouse Alexa 555 highly cross-absorbed (A21424; Thermo Fisher Scientific), and anti-rat Alexa 647 (112-606-143; Jackson).

### Quantification and statistical analysis

Quantification of fluorescence intensity and ER distribution was performed on single optical sections obtained by Airyscan imaging. Line intensity profiles across the middle of each olf hair were extracted using the PlotProfile function of ImageJ/Fiji. For each genotype, 5–7 individual hairs were analyzed. Intensity data were processed and normalized using custom Python scripts. The workflow consisted of three steps: (1) normalization of distance and fluorescence intensity values to a 0–100 scale; (2) cubic spline interpolation to generate continuous intensity curves from discrete Airyscan data points; and (3) identification of points where the mean intensity exceeded a defined threshold (typically 50 arbitrary units). These analyses were implemented using the pandas, NumPy, SciPy (CubicSpline and interp1d), and matplotlib libraries in Python 3.10.

For envelope curvature, TEM images were analyzed using Kappa in the Fiji plugin. Violin plots were generated from the median curvature values extracted from each envelope fragment. Statistical analyses were performed using the Mann–Whitney U test (two-sided), implemented in Python (SciPy).

### Cell culture experiment

*Drosophila* Schneider 2 cells were cultured in Sf-900II SFM serum-free medium (Thermo Fisher Scientific/Gibco) supplemented with penicillin and streptomycin. Cells were seeded in the 6-well plate (Iwaki). Expression vectors (pUAST-based vectors and pWA-Gal4 [Gal4 linked to the actin 5C promoter]) using TransIT-Insect Transfection Reagent (Takara) according to the manufacturer’s protocol. Forty-eight hours later, cells were reseeded onto the Con-A–coated cover glasses (15 mm) on a Parafilm in a humid chamber for 30–60 min. Coverslips were covered with 0.5 mg/ml concanavalin A (Sigma-Aldrich) in water and air-dried (Rogers et al., 2002). Cells on the cover glass were washed three times and fixed with 4% paraformaldehyde in PBS, blocked in 0.5% BSA, 0.1% Triton X-100 in PBS, and stained with the set of antibodies listed in Table S1. The FlexAble CoraLite Plus 488 Antibody Labeling Kit for Mouse IgG1 was used to label the mouse anti-Cnx antibody for double labeling with two mouse antibodies. Samples were mounted in VECTASHIELD Mounting Medium (Vector Laboratories), SlowFade Diamond Antifade Mountant (without DAPI), or ProLong Glass Antifade Mountant (without DAPI, Thermo Fisher Scientific), and images were captured with an Olympus FV1000 confocal microscope equipped with UPlanSApo 60×/1.2w and PlanApo 60×/1.40 oil objective lenses. Image deconvolution was performed by the Richardson–Lucy algorithm implemented in ImageJ. Fixed cells were regularly checked for mycobacterium contamination by DAPI staining.

### Immunoprecipitation and western blotting

Transfected cells were harvested from the culture dishes by pipetting, washed two times with PBS, and lysed in 200 μl of RIPA buffer (50 mM Tris-HCl, pH 7.5, 150 mM NaCl, 1% NP40, 0.5% deoxycholate, and 0.1% SDS, supplemented with protease inhibitor cocktail) per 35-mm well. Immunoprecipitation was performed with antibodies conjugated to magnetic beads. Western blotting was performed by electrophoresis in pre-casted acrylamide gradient gel (SuperSep Ace, Fujifilm), transferred to PVDF membrane (iBlot2 Transfer Stacks, PVDF), and incubated with antibody solutions with iBind Automated Western System (Thermo Fisher Scientific). The signal was detected with chemiluminescence (ECL Prime Reagent and Davinch-Chemisystem imager).

### Sample preparation for mass spectrometry

#### Experiment 1

S2 cells transfected with pUAST-attB HA–gox–Flag were lysed in RIPA buffer and immunoprecipitated with magnetic beads coated with anti-HA or anti-Flag antibody. Immunoprecipitates were electrophoresed in SDS-polyacrylamide gel and stained with a Silver Stain kit (Fujifilm). Each lane was cut into 16 fragments and analyzed by mass spectroscopy in the RIKEN BDR Proteomics Core facility. Proteins enriched by immunoprecipitation with anti-Flag (cytoplasmic site) but not in anti-HA (ER lumen) were identified as candidate Gox interactors.

#### Experiment 2

S2 cells co-transfected with pUAST-attB gox–Flag and pUAST-attB Ref(2)P-Myc were lysed in RIPA buffer and immunoprecipitated with magnetic beads coated with anti-Myc, anti-Flag, or anti-HA (negative control) antibody. Whole lanes of silver-stained, short-electrophoresed gel were analyzed.

#### Experiment 3

Anti-Flag immunoprecipitate of experiment 2 was electrophoresed, and the molecular mass range of 25–37 k was cut and analyzed for ubiquitinated peptides.

Gel fragments were reduced with dithiothreitol and alkylated with iodoacetamide. Digestion was carried out using MS-grade trypsin (Thermo Fisher Scientific). For ubiquitination site identification, additional digestion with pepsin (Promega) was performed. Resultant peptides were extracted using 1% trifluoroacetic acid and 50% acetonitrile solution, dried under vacuum, and reconstituted in 2% acetonitrile and 0.1% trifluoroacetic acid solution.

### LC-MS/MS analysis

In the experiment 1, mass spectra were acquired using an LTQ-Orbitrap Velos Pro (Thermo Fisher Scientific) coupled to a nanoflow UHPLC system (ADVANCE UHPLC; AMR, Inc.) with an Advanced Captive Spray SOURCE (AMR, Inc.). Peptide mixtures were loaded onto a C18 trap column (CERI, ID 0.1 × 20 mm, particle size 5 μm) and fractionated using a C18 L-column (CERI, ID 0.075 × 150 mm, particle size 3 μm). A linear gradient from 5 to 35% solvent B over 20 min at a 300 nl/min flow rate was used. Solvent compositions were 100% H_2_O with 0.1% formic acid (Buffer A) and 100% acetonitrile with 0.1% formic acid (Buffer B). The mass spectrometer operated in data-dependent mode with thirteen successive scans. Initially, full-scan MS was conducted at 60,000 resolution over the range of 350–2,000 m/z using an Orbitrap. MS/MS scans were performed on the twelve most intense ion signals using collision-induced dissociation with a normalized collision energy of 35%, a 2 m/z isolation width, and a 90-s activation time.

In the experiments 2 and 3, mass spectra were acquired using an Orbitrap Eclipse (Thermo Fisher Scientific) coupled to a nanoflow UHPLC system (Vanquish; Thermo Fisher Scientific). Peptide mixtures were loaded onto a C18 trap column (PepMap Neo Trap Cartridge, ID 0.3 × 5 mm, particle size 5 μm) and separated on a C18 analytical column (ID 0.075 × 250 mm; Aurora, particle size 1.7 μm, IonOpticks). The peptides were eluted at a flow rate of 300 nl/min using the following gradient: 0–2% solvent B over 1 min, 2–5% over 2 min, 5–16% over 19.5 min, 16–25% over 10 min, 25–35% over 4.5 min, a sharp increase to 95% over 4 min, holding at 95% for 5 min, and finally re-equilibration at 5%. The Orbitrap operated in a data-dependent mode with a 3-s cycle time. Full-scan MS was collected at 60,000 resolution, with a mass range of 375–1,500 m/z, using a standard AGC and maximum injection time of 50 ms. MS/MS scan was triggered from precursors with intensity above 20,000 and charge states 2–7. Quadrupole isolation width was 1.6 m/z, with normalized HCD energy of 30%, and resulting fragment ions were recorded in Orbitrap at 15,000 resolution with standard AGC target and maximum injection time of 22 ms. Dynamic exclusion was set to 20 s.

### Data processing

The raw data files were searched against the *Drosophila melanogaster* dataset (UniProt Proteome UP000000803) with the common Repository of Adventitious Proteins (cRAP, ftp://ftp.thegpm.org/fasta/cRAP) for contaminant protein identification, using Proteome Discoverer 2.5 software (Thermo Fisher Scientific) with the MASCOT ver.2.8 search engine, with a false discovery rate set at 0.01. The number of missed cleavage sites was set as 2. Carbamidomethylation of cysteine was specified as a fixed modification, while oxidation of methionine and acetylation of the protein N terminus were treated as variable modifications. To identify ubiquitination sites, di-glycine of lysine and deamidation of glutamine and asparagine were included as additional variable modifications.

### Online supplemental material

Fig. S1 shows FIB-SEM reconstructions of ER and plasma membrane in olf and spinule hair cells at 42 h APF. An enlarged view of the internal side of the plasma membrane is shown on the right. Fig. S2 shows autophagy gene knockdown phenotypes. Fig. S3 shows Atl RNAi phenotype. Fig. S4 shows analysis of Gox ubiquitination. Fig. S5 shows analysis of dynamin-deficient olf hair cells. Video 1 shows 3D view of the plasma membrane of the olf hair cell reconstructed from a segmented FIB-SEM stack. Table S1 shows 3D view of the plasma membrane of the olf hair cell #2 reconstructed from a segmented FIB-SEM stack. Table S2 shows list of proteins identified in the mass spectrometry analyses. Table S3 shows list of oligonucleotide primers used to construct APEX2 tag knock-in into the *gox* gene. Table S4 shows mass spectrometric identification of ubiquitination sites at K101 and K131 of Gox.

## Supplementary Material

Table S1 shows key resource table.

Table S2 shows list of proteins identified in the mass spectrometry analyses.

Table S3 shows list of oligonucleotide primers used to construct APEX2 tag knock-in into the *gox* gene.

Table S4 shows identification of ubiquitination sites at K101 and K131 of Gox. Mass spectrograms of peptide 83-LVAAPNSTDNATRPDDERKDLK-104 and 119-GLTTHTLQVNLGKLTER-135 are shown (di-glycilated lysines are bold).

SourceData FS4 is the source file for Fig. S4.

## Data Availability

Raw image data are available in the SSBD repository (https://ssbd.riken.jp/repository/375/). All mass spectrometry data have been deposited to the ProteomeXchange Consortium via jPOST with the accession numbers PXD053779 and JPST003205 (preview URL https://repository.jpostdb.org/preview/78763230166c42ea45393c, access key 9275). Original research materials (plasmid and *Drosophila* stocks) are available after signing appropriate Materials Transfer Agreements.
