# Supplementary material for: Endoplasmic reticulum patterns insect cuticle nanostructure
Source: J Cell Biol. 2025 Dec 29;225(2):e202503127. doi: 10.1083/jcb.202503127 (PMC12755865; doi:10.1083/jcb.202503127)
Supplement: Table S1 — shows key resource table. [file jcb_202503127_tables1.docx]

**Table S1.**

**Key resource table**

| REAGENT or RESOURCE | SOURCE | IDENTIFIER |
| --- | --- | --- |
| Antibodies | | |
| Rat monoclonal anti-HA (3F10, 500x) | Roche | Cat# 11988506001, RRID:AB_390916 |
| Mouse monoclonal anti-Calnexin99A (10x) | DSHB | Cat# Cnx99A 6-2-1, RRID: AB_2722011, 10x dilution |
| Rabbit polyclonal anti-RFP (200x) | MBL | Cat# PM005, RRID:AB_591279 |
| Rabbit polyclonal anti-Ref(2)P (1000x) | Y. Tamaki (Tohoku Univ.) | Nagai et al., 2021, 1000x dilution |
| anti-Flag (200) | mouse monoclonal antibody (M2) | Sigma |
| Rabbit anti-Myc-tag polyclonal Ab (200x) | MBL | 562 |
| Mouse anti-Myc-tag monoclonal Ab (My3,1000x) | MBL | M192-3 |
| Mouse anti-Multi Ubiquitin (FK2, 200x) | MBL | D058-3 |
| Goat anti-rabbit IgG (H+L) CF405S (200x) | BTI | 20082-1 |
| Goat anti-Rabbit IgG (H+L) Highly Cross-Adsorbed, Alexa Fluor Plus 405 (200x) | Invitrogen |  |
| Goat anti-rat IgG-DyLight488 AffinityPure (200x) | Jackson Immunoresearch | Discontinued? |
| Goat anti-Rabbit IgG, Alexa Fluor 488 (200x) | Thermo Fisher Scientific | Cat# A-11034, RRID:AB_2576217 |
| Goat anti-Mouse IgG Alexa Fluor 555 (200x) | Thermo Fisher Scientific | Cat# A-21424, RRID:AB_141780 |
| Goat anti-Rabbit IgG Alexa Fluor 555 (200x) | Thermo Fisher Scientific | Cat# A-21428, RRID: AB_2535849 |
| Goat anti-Rat-IgG-StarOrange (200x) | abberior | STORANGE-1007-500µG |
| Goat anti-rat IgG Alexa Fluor 647 AffiniPure (200x) | Jackson Laboratory | Cat# 112-606-143, RRID:AB_2338412 |
| Goat anti-Mouse IgG, Alexa Fluor 633 (200x) | Thermo Fisher Scientific | Cat# A-21052, RRID:AB_2535719 |
| Goat anti-Mouse-IgG-StarRed (200x) | abberior | STRED-1001-500µG |
| Anti-FLAG (M2)-HRP (500x Western) | Sigma | A8592 |
| Anti-DDDDK-tag (FLA-1GS) (PL14) -Magnetic Beads | MBL Life Science | Cat# M185-11R |
| Anti-HA-tag (TANA2)-HRP-DirecT (1000x) | MBL Life Science | Cat# M180-7 |
| Anti-HA-tag (TANA2)-Magnetic Beads | MBL Life Science | Cat# M180-10 |
| Anti-Myc-tag (PL14)-HRP-DirecT (1000x) | MBL Life Science | Cat# M047-7 |
| Anti-Myc-tag (PL14)-Magnetic Beads | MBL Life Science | Cat# M047-11 |
| Goat anti-mouse IgG-HRP (1000-5000x) | MBL |  |
| FlexAble CoraLite® Plus 488 Antibody Labeling Kit for Mouse IgG1 | Proteintech | Cat# KFA021 |
| Chemicals | | |
| Glutaraldehyde EM Grade | TAAB | Cat# G011/1 |
| 16% Formaldehyde Solution (w/v) | Thermo Fisher Scientific | Cat# 28908 |
| OsO_4_ | NissinEM | Cat# 300 |
| Sodium Cacodylate | TAAB | Cat# 3131 |
| thiocarbohydrazide (TCH) | Merck (Sigma-Aldrich) | Cat# 223220 |
| potassium ferrocyanide | nacalai tesque | Cat# 28608-42 |
| Glycine | FUJIFILM-Wako | Cat# 077-00735 |
| CaCl2 | FUJIFILM-Wako | Cat# 038-24985 |
| Acetone | FUJIFILM-Wako | Cat# 016-00346 |
| Ethanol | FUJIFILM-Wako | Cat# 057-00451 |
| PBS (10×) | Nacalai Tesque | Cat# 27575-31 |
| Bovine Serum Albumin | FUJIFILM-Wako | Cat# 017-15141 |
| Bovine Serum Albumin | MERCK/Sigma-Aldrich | Cat# A-2153 |
| DAB tablets | Merck (Sigma-Aldrich) | Cat# D5905 |
| hexamethylenetetramine (C_6_H_12_N_4_) | Merck (Sigma-Aldrich) | Cat# 13-0750 |
| silver nitrate (Ag NO_3_) | Merck (Sigma-Aldrich) | Cat# 792276 |
| tetrachlorogold (III) acid trihydrate (AuHCl_4_3H_2_O) | Merck (Sigma-Aldrich) | Cat# 520918 |
| sodium thiosulphate | Merck (Sigma-Aldrich) | Cat# 217263 |
| Sodium Tetraborate Decahydrate | FUJIFILM-Wako | Cat# 191-01425 |
| H_2_O_2_ | FUJIFILM-Wako | Cat# 081-04215 |
| Molecular Sieves 3A 1 / 16 | Nacalai Tesque | Cat# 04170-15 |
| MNA | TAAB | Cat# M012 |
| Epon812 | TAAB | Cat# T026 |
| DDSA | TAAB | Cat# D027 |
| DMP-30 | TAAB | Cat# D035 |
| L-asparatic acid | Nacalai Tesque | Cat# 03503-72 |
| KOD-ONE | TOYOBO | Cat# KMM-201 |
| EcoRI | TAKARA | Cat# 1040A |
| In-Fusion HD cloning kit | TAKARA | Cat# 639648 |
| Concanavalin A | Signa-Aldrich | C7642 |
| VECTASHIELD PLUS Antifade Mounting Medium with DAPI | Vector Laboratories | H-2000-2 |
| SlowFade Diamond Antifade Mountant | Thermo Fisher Scientific | Cat# S36972 |
| ProLong Glass Antifade Mountant (without DAPI) | Thermo Fisher / Invitrogen | P36980 |
| Sf-900II SFM | Thermo Fisher/Gibco | Cat# 10902096 |
| Penicillin-Streptomycin (10,000 U/mL) | Thermo Fisher/Gibco | Cat# 15140122 |
| Polyoxyethylene（20）Sorbitan Monolaurate ［Tween 20］ | FUJIFILM Wako Pure Chemical Corporation | Cat# 167-11515 |
| TritonX-100 | MERCK/Sigma-Aldrich | Cat# T-9284 |
| ECL™ Prime Western Blotting Detection Reagent | cytiva | Cag# RPN2232 |
| Silver Stain MS Kit | FUJIFILM Wako Pure Chemical Corporation | Cat# 299-58901 |
| WB Stripping Solution | Nacalai Tesque | 05364-55 |
| Supplies |  |  |
| SuperSep (TM) Ace, 5-20%, 17well | FUJIFILM-Wako | Cat# 194-15021 |
| SuperSep (TM) Ace, 5-20%, 13well | FUJIFILM-Wako | Cat# 197-15011 |
| iBind Flex Western Device | Thermo Fisher/Invitrogen | Cat# SLF2000 |
| iBind Flex Solution Kit | Thermo Fisher/Invitrogen | Cat# SLF2020 |
| iBind Flex Cards | Thermo Fisher/Invitrogen | Cat# SLF2010 |
| iBind Cards | Thermo Fisher/Invitrogen | Cat# SLF1010 |
| 35mm GLASS BASE DISH glass 27φ | AGC TECHNO GLASS/IWAKI | Cat# 3910-035 |
| MICROPLATE with Lid 6well | AGC TECHNO GLASS/IWAKI | Cat# 3810-006 |
| iBlot2 Dry blotting system | Thermo Fisher Scientific | Cat# IB21001 |
| iBlot2 Transfer Stacks, PVDF, regular size | Thermo Fisher Scientific | Cat# IB24001 |
| iBlot2 Transfer Stacks, PVDF, mini | Thermo Fisher Scientific | Cat# IB24002 |
| Block incubator | IWAKI Duble Aluminum Bath | Cat# ALB-301 |
| EM supplies |  |  |
| PIN stub φ12.5 x 8mm | EM Japan | Cat# G301 |
| Conductive EPOXY | Chemtronics | Cat# CW2400 |
| Experimental Models: Organisms/Strains |  |  |
| D. melanogaster: y[1] w{*} | Akira Nakamura |  |
| D. melanogaster: neur-Gal4 | Francois Schweisguth | FBti0017282 |
| *D. melanogaster: y w; gox[1]* | Ando et al. | Ando et al., 2019 |
| *D. melanogaster: y w; genomic HAgox* | Ando et al. | Ando et al., 2019 |
| *D. melanogaster: y w; neur-Gal4 gox[1]/ Tm6c Sb Dfd-GMR-YFP* | Ando et al. |  |
| *D. melanogaster: w[1118]; PBac{20XUAS-tdTomato-Sec61β}VK00037* | BDSC | 64746 |
| *D. melanogaster: y w; APEX2gox* | This work |  |
| *D. melanogaster: Osi23[KK1039329]* | VDRC | v101220 |
| *D. melanogaster: Atg8a[GD4654]* | VDRC | v43096 |
| *D. melanogaster: Atg8a[GD4654]* | VDRC | v43097 |
| *D. melanogaster: Atg1[NIG.10967R]* | NIG | 10967R-1 |
| *D. melanogaster: Atg1[GL00047]* | NIG | GL00047 |
| *D. melanogaster: Atg2[KK101148]* | VDRC | v108448 |
| *D. melanogaster: Atg2[VSH330026]* | VDRC | v330026 |
| *D. melanogaster: Atg5[NIG.1643R]* | NIG | 1643R-2 |
| *D. melanogaster: Atg9[NIG.3615R]* | NIG | 3615R-2 |
| *D. melanogaster: Atg18a[HMS01193]* | NIG | HMS01193 |
| *D. melanogaster: atlastin[GD242]* | VDRC | v6714 |
| *D. melanogaster: sponge[GD10145]* | VDRC | v21293 |
| *D. melanogaster: TER-97[GD9777]* | VDRC | v24354 |
| *D. melanogaster:CG13887[KK108479]* | VDRC | v106452 |
| *D. melanogaster: Kr-h2[GD937]* | VDRC | v7893 |
| *D. melanogaster: ref(2)P[HMS00551]* | BDSC | 36111 |
| *D. melanogaster: spg[GFP]* | Yohanns Bellaïche | Pietro et al., 2023. |
| *D. melanogaster: shibire[ts2]* | Kyoto Drosophila Stock Center | Stock # 106754, FBal0015611 |
| *D. melanogaster: UAS-shibire[ts1]* | Bloomington Drosophila Stock Center | Stock # 44222, FBti0151794 |
| *D. melanogaster: UAS-PLCdelta-PH-EGFP}3* | Bloomington Drosophila Stock Center | Stock # 39693, FBti0151794 |
| *D. melanogaster: UAS-shi-GFP* | Elisabeth Chen (UT Southwestern) | Zhang et al., 2020 |
| *D. melanogaster: mVenus-Tyn; mScarlet-Dyl* | Yuki Itakura | Itakura et al., 2024. |
| Recombinant DNA |  |  |
| pWA-Gal4 | Yasushi Hiromi | Personal communication |
| pUAST-attB HA-gox |  | Ando, et a., 2019 |
| pUAST-attB gox-Flag |  | Ando, et a., 2019 |
| pUAST-attB HA-gox-Flag |  | This study |
| pUAST-attB HA-gox[4KR]-Flag |  | This study |
| pUAST-attB HA-gox[8KR]-Flag |  | This study |
| pUAST-attB HA-gox[11KR]-Flag |  | This study |
| pUAST-attB Myc-Ref(2)P |  | This study |
| pUAST-attB Ref(2)P-Myc |  | This study |
| Cell culture |  |  |
| Drosophila Schneider 2 cells | RIKEN Bioresource Center | RCB115 |
| Sf-900II SFM 10902096 | Thermo Fisher Scientific | Cat# 10902096 |
| L-Glutamine (200 mM) | Thermo Fisher Scientific | Cat# A2916801 |
| TransIT-Insect Transfection Reagent | Takara | Cat# MIR6100 |
| 6-well culture plate | Iwaki | Cat# 3810-006N |
| Round cover glass (12mm) | Matsunami | Car# C012001 |
| Software |  |  |
| ImageJ-Fiji |  | https://fiji.sc |
| Amira (Version 2020.2) | Thermo Fisher Scientific | RRID:SCR_007353 |
| Microscope, Electron Microscope, Imaging |  |  |
| Confocal microscope | Carl Zeiss | LSM880, LSM980 Airyscan detector |
| Confocal microscope | Olympus | FV1000 |
| FIB-SEM | Thermo Fisher Scientific | Helios G4 UC |
| FIB-SEM | Thermo Fisher Scientific | Aquilos2 |
| FE-SEM | JEOL | JSM-IT700HR |
| TEM | JEOL | JEM-1400Plus |
| Osmium Coater | Meiwafocis | Tenant 20 |
| Davinch-Chemisystem | Core Biosystem Co., Ltd. | CAS-400SM |
| LC/LC Mass spectroscopy |  |  |
| LTQ-Orbitrap Velos Pro coupled to a nanoflow UHPLC system (ADVANCE UHPLC; AMR Inc.) with an Advanced Captive Spray SOURCE (AMR Inc.). | Thermo Fisher Scientific |  |
| Orbitrap Eclipse coupled to a nanoflow UHPLC system (Vanquish) | Thermo Fisher Scientific |  |
