# Supplementary material for: Endoplasmic reticulum patterns insect cuticle nanostructure
Source: J Cell Biol. 2025 Dec 29;225(2):e202503127. doi: 10.1083/jcb.202503127 (PMC12755865; doi:10.1083/jcb.202503127)

SourceDataFS4

Fig. S4A Blot: anti-Myc

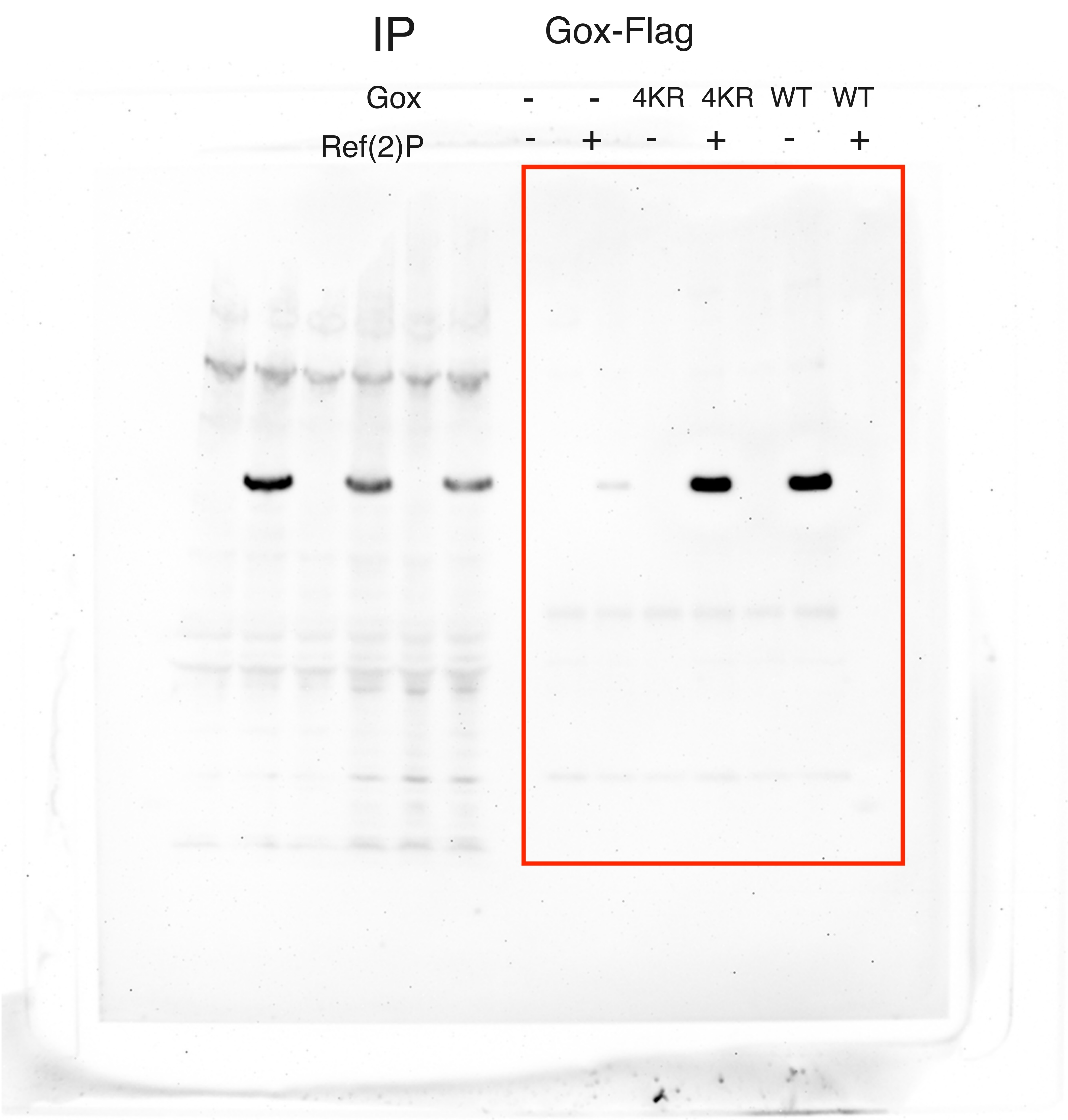

Fig. S4A Blot: anti-Flag

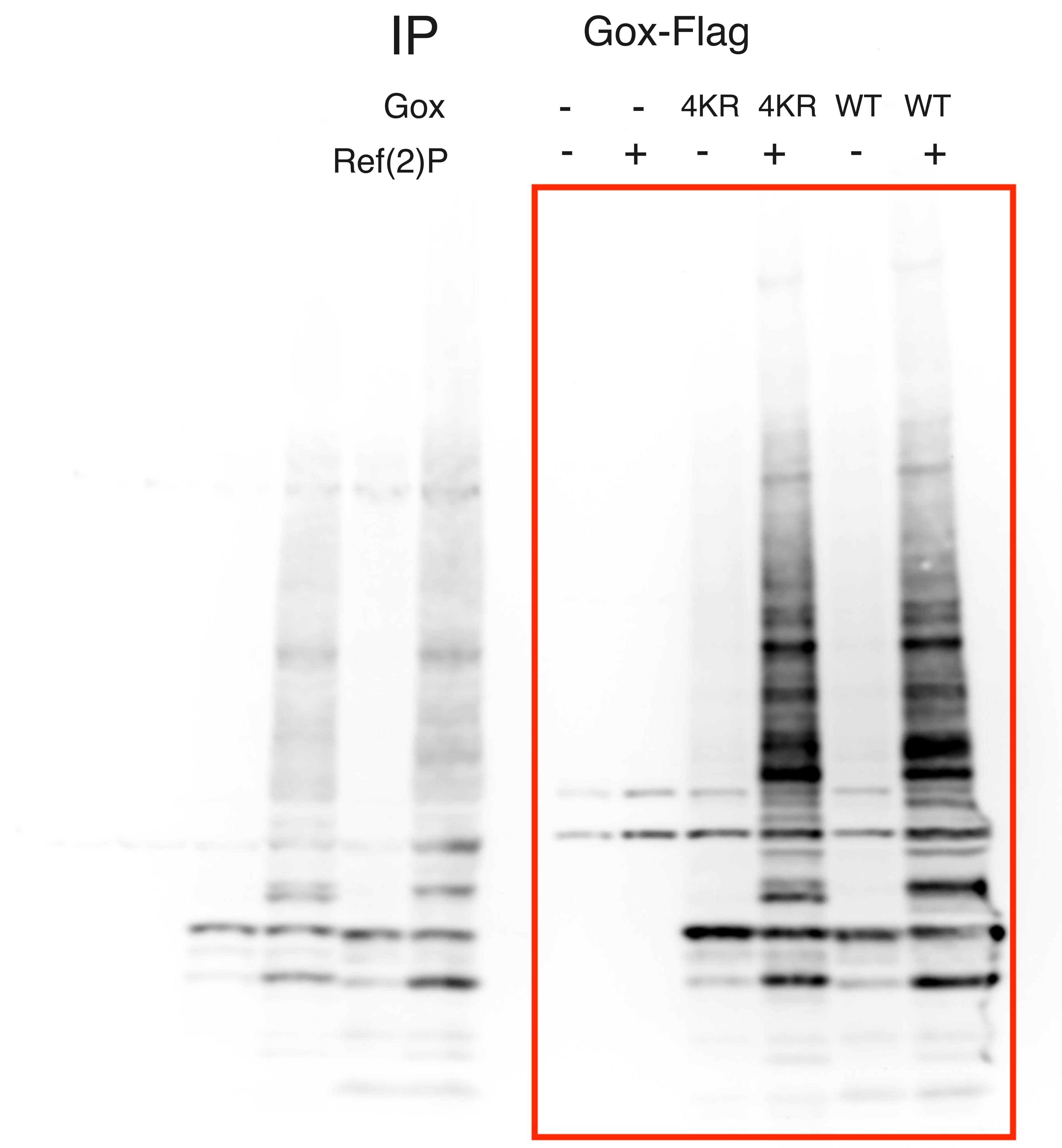

Fig. S4A Blot: anti-Ubi

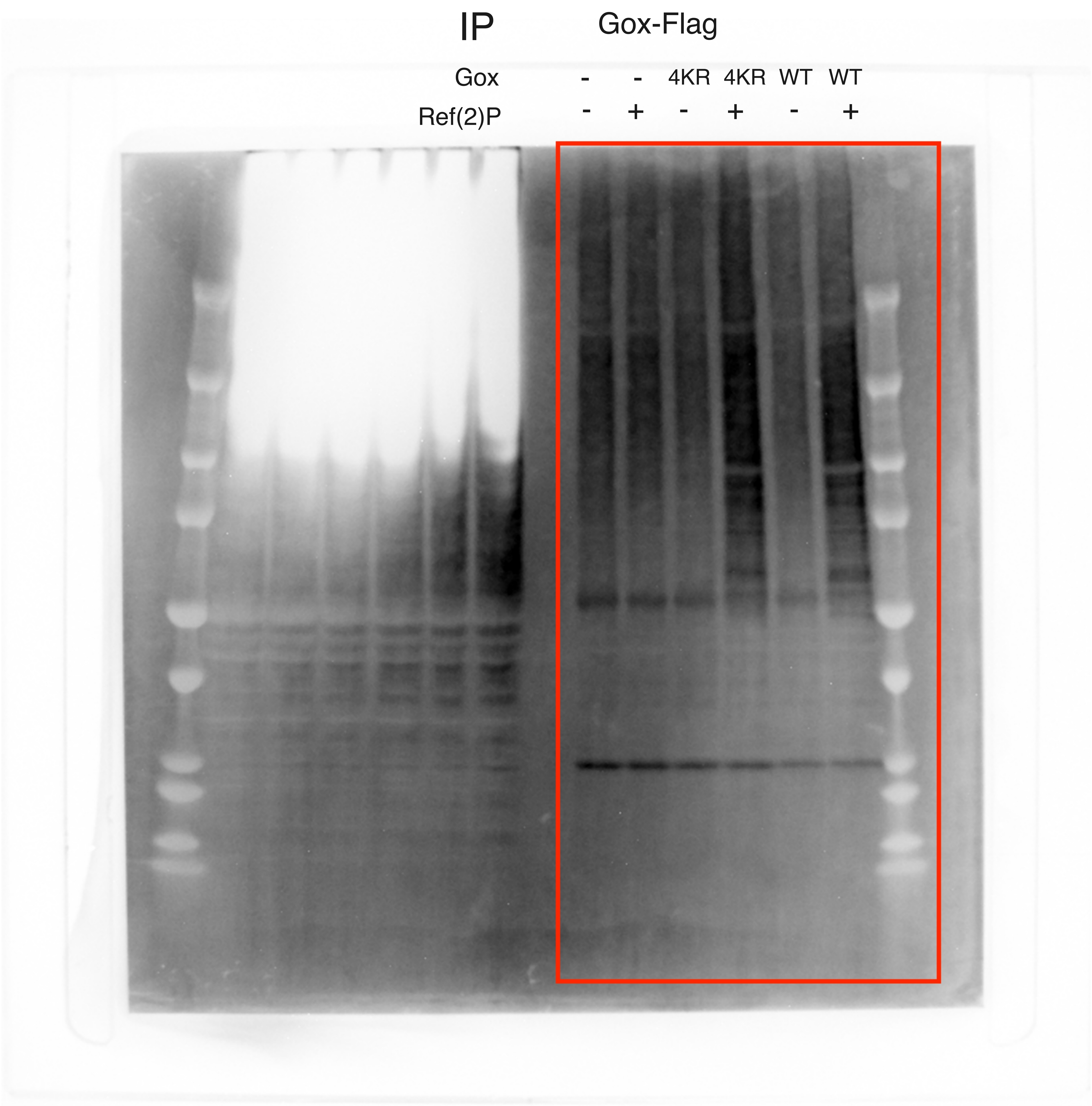

Fig. S4B Blot: anti-Myc

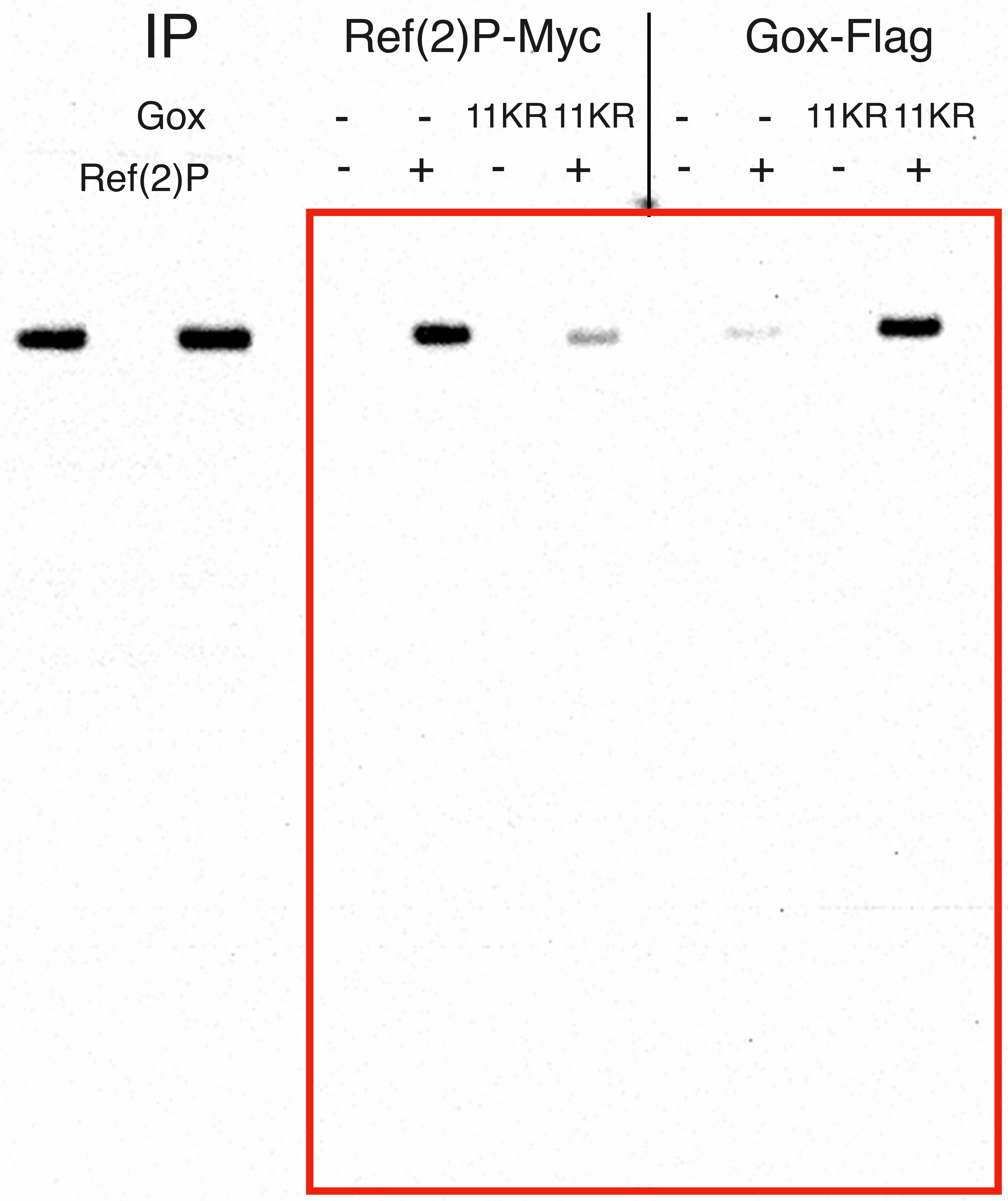

Fig. S4B Blot: anti-Flag

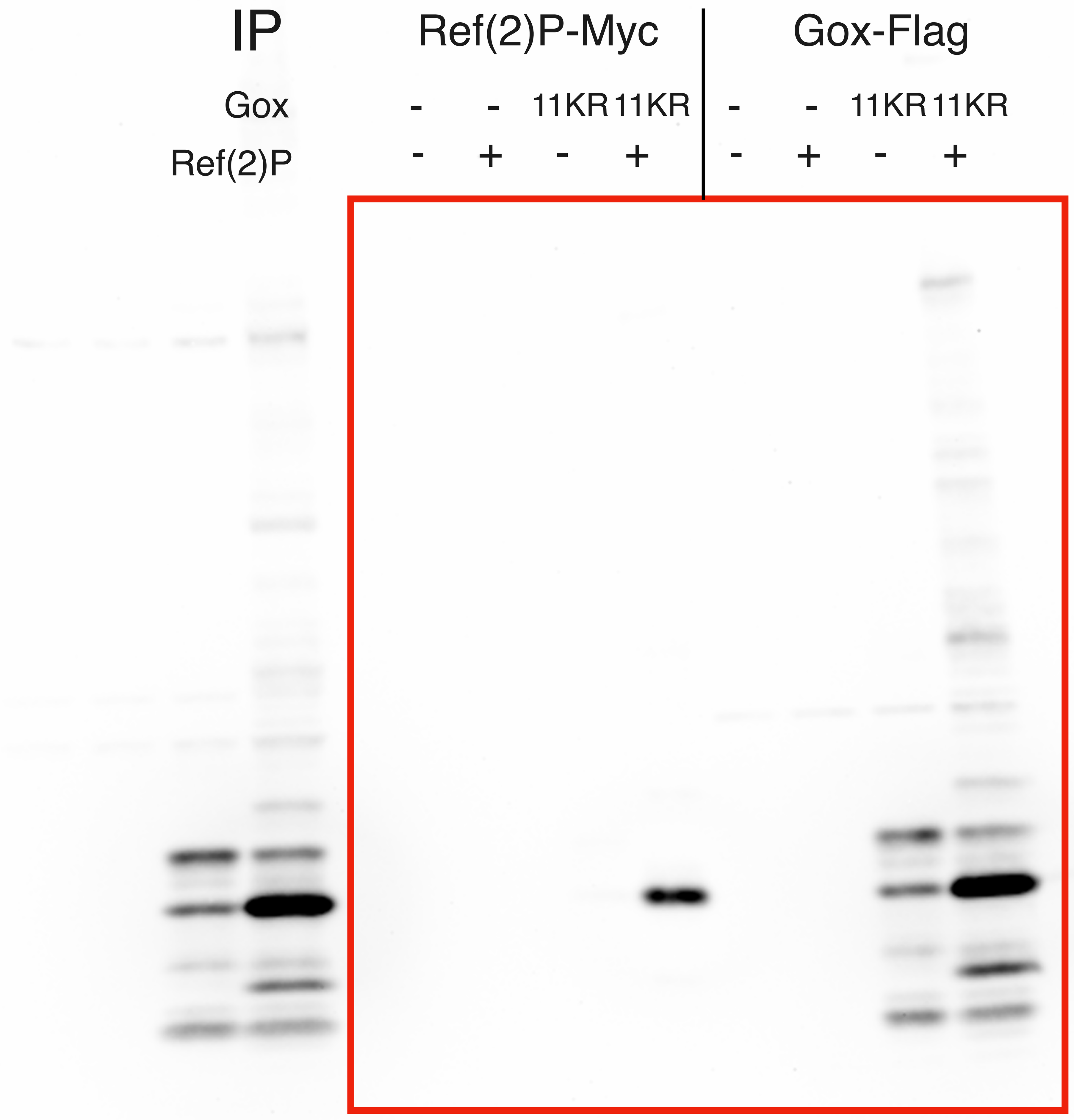

Fig. S4B Blot: anti-Ubi

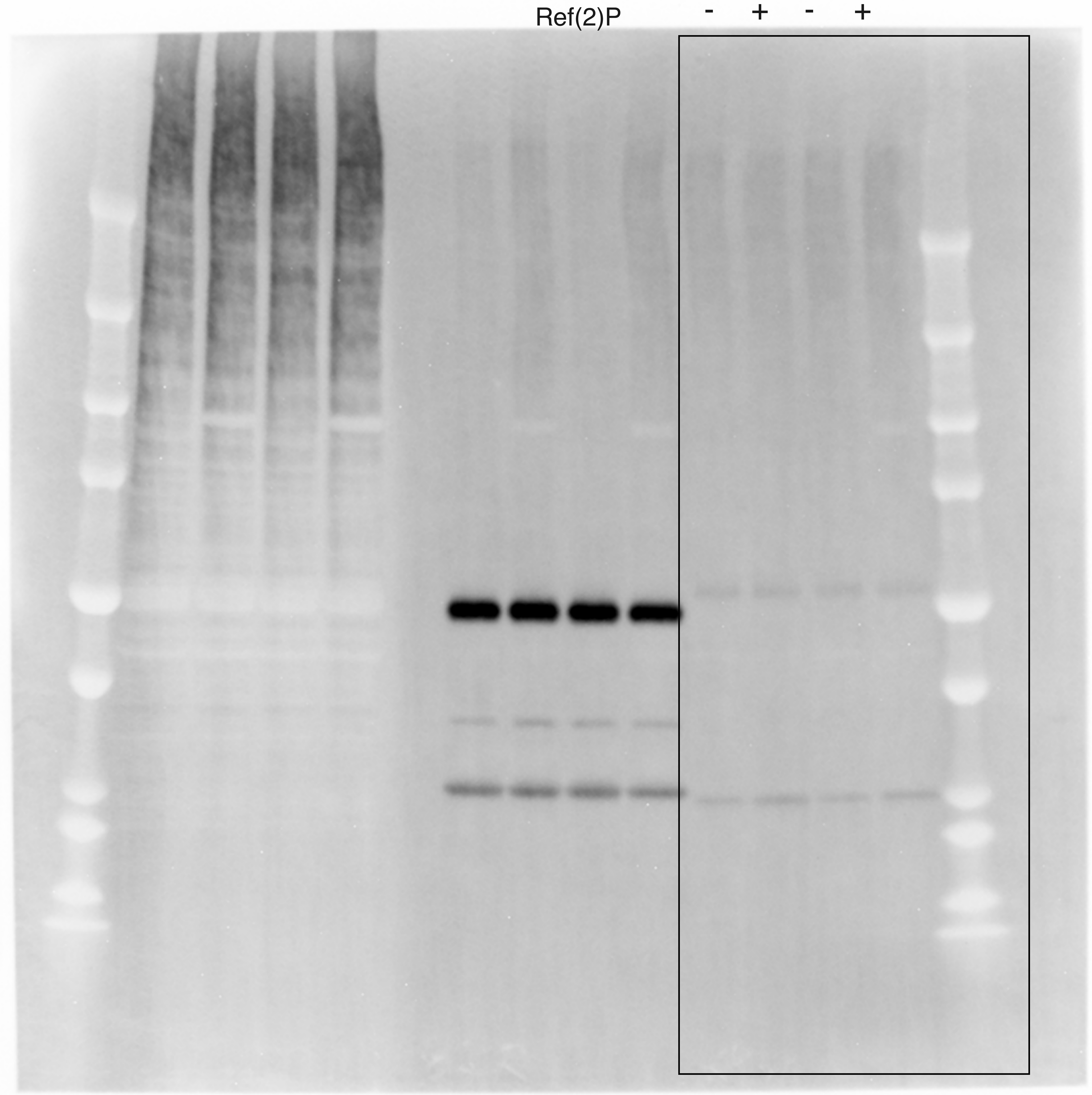

Supplement: SourceData FS4 — is the source file for Fig. S4. [file jcb_202503127_sourcedatafs4.pdf]
